# LeuO, a LysR-Type Transcriptional Regulator, Is Involved in Biofilm Formation and Virulence of *Acinetobacter baumannii*

**DOI:** 10.3389/fcimb.2021.738706

**Published:** 2021-10-11

**Authors:** Md. Maidul Islam, Kyeongmin Kim, Je Chul Lee, Minsang Shin

**Affiliations:** Department of Microbiology, School of Medicine, Kyungpook National University, Daegu, South Korea

**Keywords:** *Acinetobacter baumannii*, LeuO, transcriptome (RNA-seq), biofilm, virulence, adherence

## Abstract

*Acinetobacter baumannii* is an important nosocomial pathogen that can survive in different environmental conditions and poses a severe threat to public health due to its multidrug resistance properties. Research on transcriptional regulators, which play an essential role in adjusting to new environments, could provide new insights into *A. baumannii* pathogenesis. LysR-type transcriptional regulators (LTTRs) are structurally conserved among bacterial species and regulate virulence in many pathogens. We identified a novel LTTR, designated as LeuO encoded in the *A. baumannii* genome. After construction of LeuO mutant strain, transcriptome analysis showed that LeuO regulates the expression of 194 upregulated genes and 108 downregulated genes responsible for various functions and our qPCR validation of several differentially expressed genes support transcriptome data. Our results demonstrated that disruption of LeuO led to increased biofilm formation and increased pathogenicity in an animal model. However, the adherence and surface motility of the LeuO mutant were reduced compared with those of the wild-type strain. We observed some mutations on amino acids sequence of LeuO in clinical isolates. These mutations in the *A. baumannii* biofilm regulator LeuO may cause hyper-biofilm in the tested clinical isolates. This study is the first to demonstrate the association between the LTTR member LeuO and virulence traits of *A. baumannii*.

## Introduction

*Acinetobacter baumannii* is a member of ESKAPE pathogens that primarily affect patients with compromised defense in hospitals (Rice, 2008). Hospital-acquired *A. baumannii* infection can cause bacteremia, urinary tract infection, traumatic skin, and pneumonia (McConnell et al., 2013). Because of the nature of multidrug resistance (MDR), the World Health Organization (WHO) has listed *A. baumannii* as the “top priority” pathogen that requires new therapeutic options (WHO, 2017). *A. baumanni* can survive and persist in harsh environmental conditions in hospital settings, an ability that helps prolong outbreaks of nosocomial infection (Jawad et al., 1998). Numerous virulence factors contribute to successful *A. baumannii* infection, including biofilm formation on biological and innate surfaces (Longo et al., 2014), adherence to and invasion of host cells (Lee et al., 2006), efflux pumps that extrude different molecules and antibiotics (Kumar and Schweizer, 2005), outer membrane protein A (OmpA) that mediates interaction with epithelial cells (Lee et al., 2010), iron acquisition system (Gaddy et al., 2012), and capsular polysaccharide (Russo et al., 2010). *A. baumannii* pili are a key factor for biofilm formation, and *csuA/BABCDE* chaperone-usher secretion system-mediated pili help planktonic bacteria to adhere onto abiotic surfaces for biofilm formation (Tomaras et al., 2003). Besides abiotic surfaces, *A. baumannii* can attach onto biotic surfaces such as respiratory epithelial cells, which is another important virulence factor for infection (Lee et al., 2008). Quorum sensing in *A. baumannii* is another important pathway by which the pathogen senses extracellular signals and regulates biofilm formation and virulence (Bhargava et al., 2015). However, understanding the molecular mechanisms of virulence factors would help develop novel strategies to prevent multidrug-resistant *A. baumannii* infection.

Transcriptional regulatory proteins help prokaryotes to communicate between environmental conditions and DNA transcription to survive in different habitats (Santos et al., 2009). Bacterial genomes encode several transcriptional regulatory proteins required for adaptive cellular responses belonging to different families, such as ArsR, AsnC, DeoR, GntR, IclR, LacI, LuxR, XylS, MarR, MerR, NtrC, TetR, YedF, and YhdG. Among these, the family of LysR-type transcriptional regulators (LTTRs) is the largest and resemble approximately 16% of the overall transcriptional factors in bacteria (Srinivasan et al., 2013). A typical LTTR comprises an N-terminal DNA-binding helix-turn-helix (HTH) domain and a C-terminal coinducer-binding domain (also known as a regulatory domain). LTTRs can function as either an activator or repressor of single or operonic gene expression, which is why they have been recently termed as global transcriptional regulators (Schell, 1993). LTTRs are associated with the control of various cellular processes. For instance, VirR in *Rhodococcus equi* and MvfR and PA2206 in *Pseudomonas aeruginosa* are involved in virulence and quorum sensing (Russell et al., 2004; Deziel et al., 2005). Moreover, the proteins CidR in *Staphylococcus aureus* and OxyR in *Klebsiella pneumoniae* are involved in antibiotic resistance (Rice et al., 2005; Yang et al., 2005). In *Yersinia pseudotuberculosis*, RovM controls cell invasion, motility, and virulence (Heroven and Dersch, 2006).

LeuO is a member of the LysR family of transcriptional regulators, and members of this family have been investigated in several bacteria, including *Escherichia coli*, *Salmonella enterica*, *Vibrio cholerae*, *Yersinia enterocolitica*, and *Enterobacter cloacae* (Guadarrama et al., 2014). LeuO controls several biological functions such as biofilm formation and virulence in *V. cholerae* and *E. coli*, regulates the expression of OmpS1 and OmpS2, and downregulates the expression of OmpX, which alter the transport of hydrophobic compounds and virulence in *S. enterica* (Moorthy and Watnick, 2005; Hernández-Lucas et al., 2008; Shimada et al., 2011). LeuO regulates a wide variety of genes that are involved in amino acid biosynthesis, nitrogen fixation, quorum sensing, and virulence (Schell, 1993). It also regulates bile tolerance, antibiotic resistance, and promoter binding in *V. cholera* (Bina et al., 2016). However, LeuO has not yet been characterized in *A. baumannii* and its functions also remain unclear.

In this study, to further understand the role of LeuO in *A. baumannii*, we generated a knockout mutant of LeuO and conducted transcriptome analysis to compare the differentially expressed genes between ΔLeuO and wild-type strains. Transcriptome analysis showed that several biological and metabolic pathways are altered after the deletion of LeuO. Our experiments on biofilm formation, surface motility and adherence to epithelial cell suggested that some genes related to these features are directly or indirectly regulated by LeuO. Overall, our study results provide novel understanding about the regulatory role of LeuO and the pathogenesis of *A. baumannii*. This identification of the role of transcriptional regulators may help in the development of novel therapeutics against MDR *A. baumannii* strains.

## Materials and Methods

### Bacterial Strains, Plasmids, and Culture Conditions

*A. baumannii* ΔLeuO and complementation strains were constructed using the homologous recombination method as described in the supplementary data. *A. baumannii* strains were grown in Luria–Bertani (LB) media at 37°C or 30°C, and agar was added at the indicated concentrations obtained from Difco or Eiken (Eiken Chemical, Tokyo, Japan). Chloramphenicol (20 µg/mL), kanamycin (50 µg/mL), and ampicillin (100 µg/mL) were added to LB broth or LB agar plates to maintain the plasmids in *E. coli*. The bacterial strains, plasmids, and primers used in this study are listed in **Supplementary Tables S3** and **S4**.

### Isolation of Bacterial mRNA and RNA Sequencing

Overnight bacterial cultures of both *A. baumannii* ATCC 17978 wild-type and ΔLeuO strains were subcultured in 10 mL of LB by 1:100 dilution and grown at 37°C until OD_600_ reached 1.00 under shaking condition. Total RNA was extracted using Qiagen RNeasy Mini kits (Qiagen, Hilden, Germany) according to the manufacturer’s instructions. The total RNA concentration was measured using the NanoDrop 2000 Spectrophotometer (Thermo Fisher Scientific). Two biological replicates of each were sent to Macrogen Inc. (Seoul, Republic of Korea), where mRNA quality control (QC), cDNA library preparation, library QC, template preparation, template QC, and RNA sequencing were performed on the Illumina NovoSeq 6000 platform. RNA-sequencing reads were aligned to the *A. baumanni* strain ATCC 17978 (GCF_ 0000 15 425.1 _ A S M1542v1). Bowtie 1.1.2 (http://bowtie-bio.sourceforge.net/index.shtml) and HTSeq version 0.10.0 software (http://www-huber.embl.de/users/anders/HTSeq/doc/overview.html) were used for analyzing the sequencing data. Any of the sequencing reads with a fold change of <2 and a p value of >0.05 were eliminated.

### Quantitative Real-Time PCR for RNA-seq Data Validation

Total RNA from *A. baumannii* strains was isolated as described earlier, and cDNA was synthesized using the M-MLV cDNA Synthesis kit (Enzynomics). Real-time PCR amplification of cDNA was performed using the ABI Step One Plus Real-Time System (Applied Biosystems), and TOPreal™ q-PCR 2X PreMIX (SYBR Green with high ROX, Enzynomics) was used. The internal forward and reverse primers used in this study for each gene are listed in **Supplementary Table S4**. The expression level was standardized relative to the transcription level of 16S rRNA expression level. The fold change was determined using the ΔΔCt method. Experiments were performed in three independent replicates.

### Biofilm Formation Assay

Biofilm formation by *A. baumannii* strains was evaluated according to the method described by (Stepanovic et al. (2000) with some modifications using a crystal violet staining assay. Briefly, bacterial strains were cultured overnight, resuspended in fresh LB broth without salt, and adjusted to a turbidity of 1.0 at 600 nm using a spectrophotometer. After dilution, the bacterial suspensions were aliquoted as 2 ml each into 5-mL polystyrene tubes and incubated for 24 h at 30°C under static conditions in a dark room. After the removal of supernatants, the tubes were washed twice with 2 ml distilled water to remove planktonic or loosely adherent cells. After air-drying the tubes for 10 min, 2 mL of crystal violet (0.1% v/v) was added to each tube to stain the inner wall with biofilm for 15 min. The stained biofilms were solubilized with 2 mL of 95% ethanol for 10 min, and 200 μL of each sample was transferred to a 96-well plate to measure turbidity at 570 nm using a microplate reader (Molecular Devices, Sunnyvale, USA.). To compensate for growth differences, turbidity was also measured at 600 nm before staining the biofilm. Three independent experiments were performed, each in triplicate.

Biofilm formation assays using *A. baumannii* 17978 wild-type strain, ΔLeuO strain, and clinical isolates of various sequence types (ST-208, ST-229, ST-357, ST-451, ST-552, and ST-784) were also performed as described earlier with two independent experiments.

### Pellicle Formation Assay

Pellicle formation assay was performed based on a method described by Martí et al. (2011). Bacterial strains were cultured overnight in LB broth without salt and diluted at 1:40 with the same media. The assay was performed in 5-ml polypropylene tubes, and 2 ml of bacterial suspensions was aliquoted into each tube with incubation at 30°C for 48 h without shaking. The pellicle film was isolated from the tube by adding 1 ml methanol. The pellicle biomass was measured (optical density at 600 nm [OD_600_]) after resuspending the pellets in 1 ml PBS. Experiments were performed in triplicates.

### Surface Motility Assay

Motility assays were performed as described previously (Clemmer et al., 2011) with some modifications. Modified LB agar containing Eiken agar 3 g/L, tryptone 10 g/L, and yeast extract 5 g/L was autoclaved and cooled at 60°C. Modified LB medium was poured into Petri dishes, dried for 8 h, and used on the same day of preparation. For testing motility, *A. baumannii* strains were grown overnight and adjusted to the same optical density (OD_600_ = 1.0) by adding modified LB broth, and then 2 µL of culture was inoculated onto the center of agar plates. The plates were incubated at 37°C for 10 h, after which the diameter of motility zones was measured. The plates were photographed using a digital imaging system. Assays were performed in triplicate with three biological replicates each time.

### Adherence Assay

Bacterial adherence to A549 cells was evaluated as described previously (Lee et al., 2006). Briefly, A549 human alveolar epithelial cells were grown in RPMI 1640 medium (HyClone, Logan, UT) supplemented with 10% heat-inactivated fetal bovine serum (HyClone), 100 U/ml penicillin G, and 50 μg/ml streptomycin. Cultures with 80%–90% confluency were trypsinized and seeded at a density of 2 × 10^5^ cells/ml in 6-well culture dishes to obtain a monolayer. After 24-h incubation, the cells were washed twice with PBS and incubated with RPMI 1640 medium without antibiotics. *A. baumannii* strains were grown to reach an OD A_600_ of 1.0 and suspended in the same media. Then, 2 × 10^7^ CFU/ml of bacteria were added into each well to obtain a multiplicity of infection (MOI) of 1:100 and incubated for 2 h at 37°C. To determine bacterial adhesion, cells were washed five times with PBS and lysed with 0.1% Triton X-100 at 37°C for 20 min. Colony-forming units were counted after 10-fold serial dilution of lysate samples to determine the number of bacteria that had attached to or invaded the A549 cells. Adherence assays were performed in three independent replicates.

### *In Vivo* Virulence Assay

All animal infection experimental procedures were approved by the Animal Care Committee of Kyungpook National University, South Korea (approval number: KNU-2019-178). Briefly, 8-week-old female BALB/c mice were maintained under conventional conditions at five mice per case and allowed access to food and water throughout the experiment. To promote infection, neutropenic mice were induced by intraperitoneal (IP) injection of cyclophosphamide (150 mg/kg) in PBS before bacterial infection (−4 and −1 day). *A. baumannii* 17978 WT, ΔLeuO, CP, and *A. baumannii* 1656-2 WT strains were grown overnight in LB broth at 37°C, washed with PBS, and the concentration was set to 2 × 10^9^ CFU/ml. Mice were injected intraperitoneally with 100 µl PBS (control) and *A. baumannii* strains (1 × 10^8^ CFU/ml) per mice (n = 5 per group). Animals were monitored every 12 h over a period of 4 days. The number of live and dead animals was input into GraphPad Prism, and survival curve was generated. Statistical analysis was conducted using the Kaplan–Meier test in GraphPad Prism.

## Results

### *A. baumannii* A1S_1874 Is the LTTR LeuO

**Figure 1:**
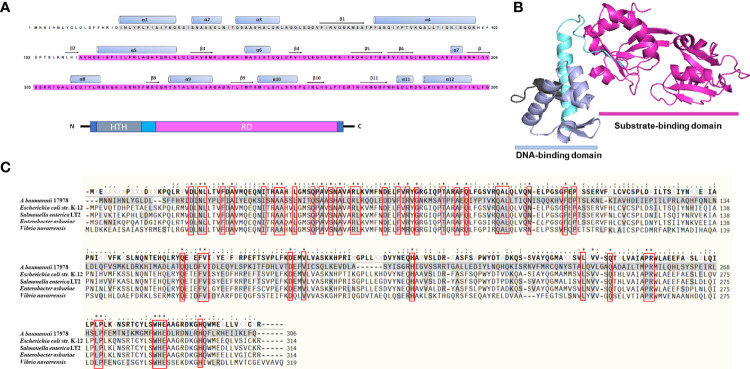
Predicted three-dimensional structure and sequence alignment of LeuO. **(A)** Deduced amino acid sequence of *A1S_1874* where the ash box indicates the helix-turn-helix DNA-binding domain (25–79) and the purple box indicates the LysR family substrate-binding domain (112–304). **(B)** Predicted 3D structure of *A1S_1874* obtained using protein modeling server Phyre2 and PyMoL program. **(C)** Multiple sequence alignment using COBALT: Multiple Alignment Tool of *A*. *baumannii A1S_1874* with previously characterized LTTR member LeuO from *E. coli* strain *K-12*, *Salmonella enterica LT2*, *Enterobacter asburiae*, and *Vibrio navarrensis*. “*”, “.”, “:” indicate most conserved residues and semi-conserved sequence, respectively.

LTTRs are organized as an N-terminal HTH DNA-binding domain and a C-terminal effector-binding domain (EBD) connected by a long linker helix (Muraoka et al., 2003). *A. baumannii A1S_1874* constitutes 306 amino acid residues, and an analysis of the amino acid sequence revealed a DNA-binding domain (HTH, 25–79) and a substrate-binding domain (112–304), indicating that A1S_1874 is a member of the LTTR family (**Figure 1A**). Blast search of the *A1S_1874* amino acid sequence (306 aa) was performed to identify the homology sequence of *A1S_1874* among other *Acinetobacter* strains and other bacterial species. The amino acid sequence was conserved among other sequenced *A. baumannii* strains. Predicted 3D structure of *A1S_1874* elucidated using the Phyre2 and PymoL software program clearly displayed two distinct domains (**Figure 1B**), which support that *A1S_1874* is a putative LTTR. Finally, we compared the amino acid sequence of *A. baumannii A1S_1874* and other gram-negative bacterial LeuO whose functions have already been characterized. The amino acid sequence of *A1S_1874* exhibited 25% sequence homology with *E. coli* LeuO, 24% sequence homology with *S. enterica* LeuO, and 25% sequence homology with *V. navarrensis* LeuO. *A. baumannii A1S_1874* demonstrated high sequence homology at the N-terminal region with other bacterial LeuO protein compared with that at the C-terminal region (**Figure 1C**). LTTRs exhibit low sequence identity among the family members, possibly due to distinct effector recognition. However, the N-terminal region displayed more sequence conservation than the C-terminal region (Schell, 1993). Considering these findings, we predict that *A1S_1874* is LeuO, an LTTR in *A. baumanni*, whose functions must be explored.

### Transcriptome Analysis of LeuO Mutant Strain

**Figure 2:**
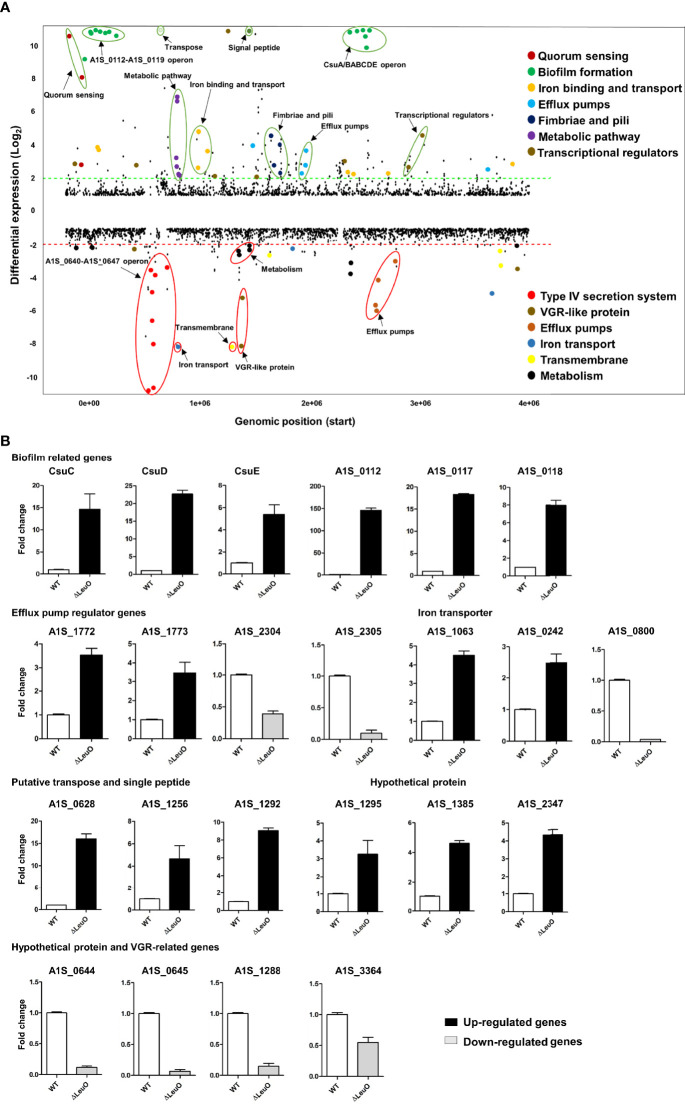
Overview of transcriptional differences between ΔLeuO and ATCC 17978 wild-type *A*. *baumannii* strains and qRT-PCR validation. **(A)** Comparative transcriptomics of *A. baumannii* ATCC 17978 and ΔLeuO strain are displayed as the differential expression. Differential expression levels are presented as fold change (mutant/wild-type) in the Y-axis, and more than 10-fold expression levels are presented above the 10-scale. Each dot indicates the differential expression levels of all predicted open reading frames of the ATCC 17978 genome and sorted according to the locus tag on the X-axis. The dash lines indicate 2-fold differential expression; upregulated and downregulated genes are located above the green line and below the red line, respectively. Gene names or functions of various highly differentially expressed genes are indicated as colored dots and circles and grouped according to functions on the right side. **(B)** qRT-PCR analysis of selected differentially expressed genes categorized as different functional groups such as biofilm-related genes, efflux pump regulator genes, iron transporter, putative transpose and signal peptide, hypothetical protein genes and VGR-related genes. Upregulated genes are presented as black-colored bars, and ash-colored bars represent downregulated genes. The data represent mean ± standard deviation from three biological replicates.

To characterize LeuO regulation in *A. baumannii* ATCC 17978, we conducted transcriptome profiling of LeuO mutant strain and compared with wild-type strain to obtain insights into the global transcriptomic changes caused by LeuO deletion. The LeuO mutant strain was constructed as described by Jung et al. (2020) (Supplementary data section), and it was confirmed using PCR analysis (**Supplementary Figure S1**). For RNA sequencing, we extracted RNA from cells growing up to an OD_600_ = 1. Differentially expressed genes were selected when the fold change in expression was ≥2 with p values <0.05 (**Figure 2A**). We found that a total of 302 genes were differentially expressed, among which 194 were upregulated and 108 were downregulated compared with those of *A. baumannii* WT and LeuO mutant. The complete list of differentially expressed genes is shown in **Supplementary Table S1**. An acyl carrier protein (locus tag *A1S_0114*) was the highest upregulated differentially expressed gene (fold change 201.60), and a hypothetical protein (locus tag *A1S_0645*) was the highest downregulated gene (fold change −16.02). In *A. baumannii* ATCC 17978, the *A1S_0112-A1S_0119* cluster is the polycistronic operon responsible for biofilm formation and virulence. Our transcriptome data indicated that the *A1S_0112-A1S_0119* cluster was highly upregulated (9.5- to 201.60-fold) in the absence of LeuO. *Csu* operon (CsuA/BABCDE) genes (*A1S_2213-2218*) were also upregulated in the LeuO mutant strain (10.40- to 64.16-fold). Proteins encoded by the *csu* operon have been identified in pellicle and biofilm formation. Quorum sensing–related genes (*A1S_0109-A1S_0111*) were also upregulated after LeuO mutation. Several genes related to iron ion binding and transport such as *A1S_0242*, *A1S_0243*, *A1S_0980*, *A1S_0981*, *A1S_1063*, and *A1S_3369* were also upregulated after LeuO mutation. Some efflux pump–related genes such as the MFS family transporters *A1S_1440* and *A1S_1772* and the RND family transporters *A1S_1649* and *A1S_1773* were also upregulated. Transcriptional regulators of different families such as the GntR family (*A1S_0072*), TetR family (*A1S_0548*), and AsnC family (*A1S_1090*) and another transcriptional regulator (*A1S_1256*) were also overexpressed. Interestingly, the gene cluster *A1S_0640-A1S_0647* (putative hypothetical protein) was highly downregulated (−3.53- to −16.02-fold) in the LeuO mutant strain. VGR-like proteins are putative T6SS effectors in *A. baumannii* that regulate cell invasion. The expression of several VGR-like protein genes (*A1S_1288*, *A1S_1289*, and *A1S_3364*) was downregulated. Iron-storing bacterioferritin (*A1S_0800* and *A1S_3175*) and several efflux pump transporters, especially RND efflux, were downregulated. Numerous genes involved in metabolism such as dehydrogenase, hydrolase, and hydratase were also downregulated. Several genes were classified as hypothetical proteins whose functions are unknown in *A. baumannii*.

We performed qPCR using the same RNA sample to validate differential gene expression levels obtained from RNA-seq. A total of 23 genes from different functional groups were selected for qPCR, including members of *A1S_0112-A1S-0119* operon, *csu* operon, efflux pump regulator, iron transporter, putative transpose and signal peptide, hypothetical proteins and VGR-like proteins (**Figure 2B**). The expression profiles were found to be consistent with data obtained from RNA-seq experiments. In some cases, there was a fold change difference in qPCR and RNA-seq data, which may be due to differences in sensitivity and specificity between the two technologies.

### Contribution of LeuO to Biofilm Formation and Pellicle Formation

**Figure 3:**
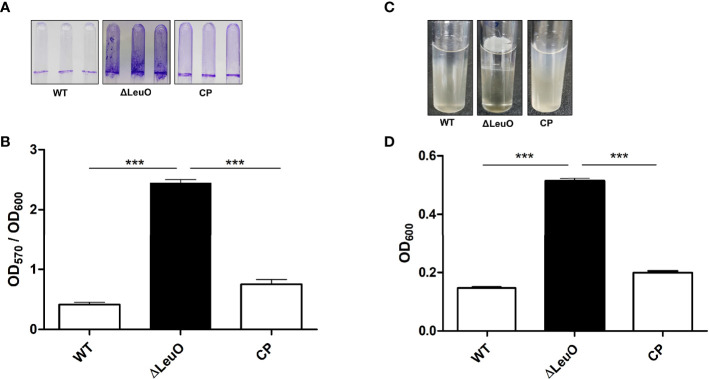
Biofilm and air–liquid interface pellicle formed by *A. baumannii* 17978 WT, *Δ*LeuO, and complementation strains. *A. baumannii* ATCC 17978 WT, ΔLeuO, and its CP strains were cultured in 5-ml polystyrene tubes at 30°C for 24 h in LB broth without salt. **(A)** Biofilm formation on polystyrene tubes was photographed after staining with 0.1% crystal violet. **(B)** Biofilm values (OD_570_) were normalized by growth levels (OD_600_) to compensate for the levels of biofilm formation on the polystyrene surface. Biofilm formation assays were conducted in triplicate, and average values of three replicates were plotted with standard deviation. **(C)** *A. baumannii* strains were cultured at 30°C for 48 h in LB broth without salt, and the pellicle formed by each strain was photographed. **(D)** Pellicles were separated by adding methanol, and pellicle biomass was measured as OD_600_. This experiment was performed in triplicate. Data are expressed as average values of three replicates with standard deviation. ***P < 0.001, significantly higher than that of the wild-type strain.

Biofilm formation is an important virulence factor for *A. baumannii* persistent infection. We conducted biofilm formation assay using the crystal violet staining method to determine the effect of LeuO deletion on the biofilm formation ability of *A. baumannii* on abiotic surfaces. The ΔLeuO strain produced significantly more biofilm than that produced by the wild-type strain. The LeuO-complemented (CP) strain yielded less biofilm than the mutant strain (**Figure 3A**). The biofilm mass formed by each strain was measured using absorbance at 570 nm of retained crystal violet, which was normalized relative to the growth of each strain using absorbance at 600 nm. The ΔLeuO strain demonstrated approximately 6-fold more biofilm than that of the wild-type strain, and CP restored the biofilm formation (**Figure 3B**). *A. baumannii* can form pellicles at the air–liquid interface. We analyzed the role of LeuO in pellicle formation using the *A. baumannii* strains in modified Luria-Bertani (LB) broth. During static culture, the ΔLeuO strain formed significantly more pellicles than the WT strain as photographed using a digital imaging system (**Figure 3C**). The pellicle biomass was measured at OD_600_ for quantification, wherein it was 0.51 for the ΔLeuO strain and 0.14 for the WT strain (**Figure 3D**). The complementation strain restored the pellicle formation to almost the same level as that of the wild-type strain.

Biofilm formation in *A. baumannii* is regulated by several specific genes, including *csuA/BABCDE*, *ompA*, *abaI*, and *pgaABCD* (Longo et al., 2014). We performed qPCR using the WT and ΔLeuO strains to determine the role of LeuO in the expression of *Csu* operon genes and thus in biofilm formation. Our results revealed that *CsuC*, *CsuD*, and *CsuE* showed 14-, 22-, and 5-fold higher expression levels, respectively, in the LeuO mutant strain than those in the wild-type strain (**Figure 2B**). We also conducted qPCR of several genes from the *A1S_0112-A1S_0119* cluster, which are responsible for biofilm formation. We observed that *A1S_0112*, *A1S_0117*, and *A1S_0118* exhibited 146-, 18-, and 8-fold higher expression levels, respectively, in the LeuO mutant strain than those in the wild-type strain (**Figure 2B**). These findings suggest that LeuO regulates the expression of biofilm-related genes and LeuO mutation results in high biofilm formation in *A. baumannii*.

### Point Mutations in LeuO Contribute to Biofilm Formation of *A. baumannii* Clinical Isolates

**Figure 4:**
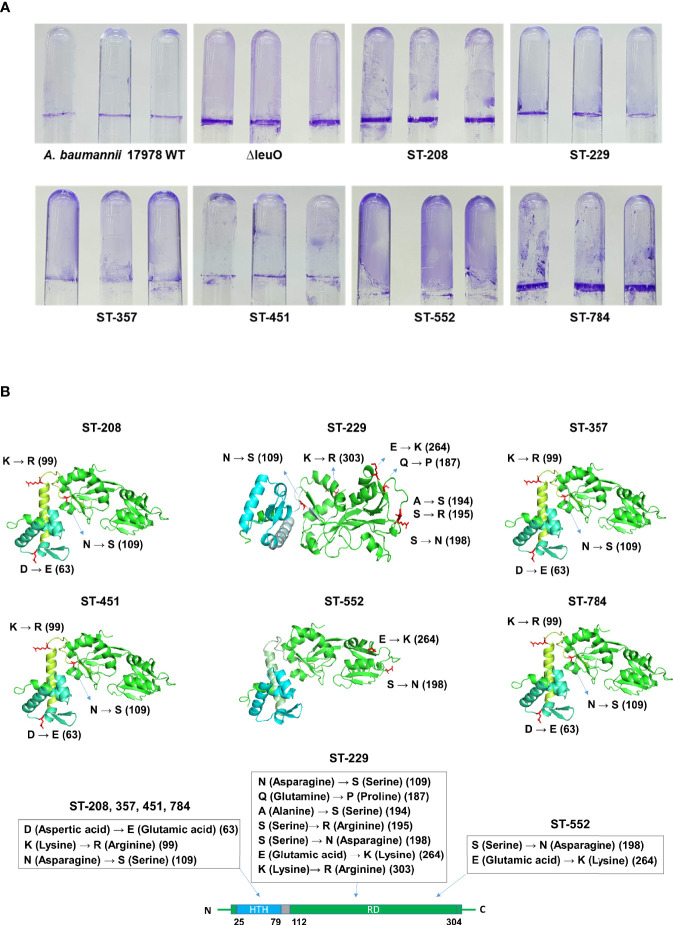
Hyper-biofilm-forming clinical strains have point mutation in the LeuO gene locus. **(A)** *A. baumannii* ATCC 17978 WT, ΔLeuO, and clinical strains of different sequence type ST-208(011), ST-229(079), ST-357(004), ST-451(001), ST-552(015), and ST-784(001) were cultured in 5-ml polystyrene tubes at 30°C for 24 h in LB broth without salt. **(A)** Biofilm formation on polystyrene tubes was photographed after staining with 0.1% crystal violet. **(B)** Cartoon representations of predicted LeuO gene structures of the clinical strains ST-208, ST-229, ST-357, ST-451, ST-552, and ST-784, respectively, obtained using the PyMoL software. Blue color indicates the N-terminal HTH domain, and green color indicates the C-terminal regulatory domain. Mutated residues are shown in red sticks at different positions such as 63, 99, 109, 187, 194, 195, 198, 264, and 303 and are marked beside.

Considering the significant regulatory role of LeuO in the biofilm formation of *A. baumannii* ATCC 17978 strain, we conducted biofilm formation assay using different clones of *A. baumannii* clinical isolates (ST-208, ST-229, ST-357, ST-451, ST-552, and ST-784). We observed that clinical *A. baumannii* isolates formed much more biofilm than ATCC 17978 strain and almost similar biofilm to that formed by the ΔLeuO strain in some cases (**Figure 4A** and **Supplementary Figure S3B**). We focused on the genetic analysis of LeuO locus (*A1S_1874*) in our tested strains to identify the cause of high biofilm formation. Sequencing analysis of LeuO locus revealed that each of the clinical isolates had 306 amino acid residues identical to those in the ATCC 17978 strain but carried several point mutations in the linker helix or in the EBD (**Supplementary Figure S3A**). In ST-208, ST-451, ST-357 and ST-784 isolates, D-to-E, K-to-R, and N-to-S point mutation changed the amino acid aspartic acid to glutamic acid, lysine to arginine, and asparagine to serine at positions 63, 99, and 109, respectively (**Figure 4B**). The isolate ST-229 exhibited a seven-point mutation at positions 109, 187, 194, 195, 198, 264, and 303 compared with that in the ATCC 17978 strain. The clinical isolate ST-552 shared two-point mutations in the regulatory domain at positions 198 and 264, which changed serine to asparagine and glutamic acid to lysine, respectively (**Figure 4B** and **Supplementary Figure S3A**). These mutations in the *A. baumannii* biofilm regulator LeuO may modulate LeuO stability and cause hyper-biofilm formation in the tested clinical isolates.

### Contribution of LeuO to Surface Motility

**Figure 5:**
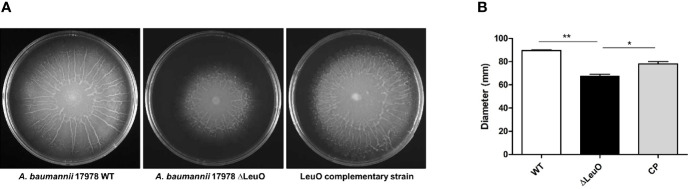
Comparison of surface motility of *A. baumannii* 17978 WT, *Δ*LeuO, and complementation strains. **(A)** 0.3% Eiken soft agar plates were used to test the surface motility of *A. baumannii* strains. Migration of bacteria from the inoculation point was photographed after culturing at 37°C for 10 h. **(B)** Average area of migration (diameter in mm) of three biological replicates is presented with standard deviation. The experiment was performed in triplicates. *P < 0.05, **P < 0.01, significantly lower than that of the wild-type strain.

We next determined whether there was any influence of LeuO on surface motility by comparing motility with that of wild-type parent strain, ΔLeuO strain, and complementation strain on semisolid motility agar plates. Bacterial migration from the center of agar plates was measured at a point of time. Migration distance of the ΔLeuO strain from its inoculating point was smaller than that of the wild-type strain (68 mm in the mutant and 89 mm in the wild-type strain; (**Figures 5A, B**). The impaired motility of the ΔLeuO strain was restored in the complementation strain. Comparative transcriptome analysis revealed that several type VI pili genes (VGR-like proteins) were downregulated in the LeuO mutant strain compared with those in the wild-type strain. VGR-like protein genes were validated by qPCR (**Figure 2B**), which illustrated decreased expression of those genes in the LeuO mutant strain. These results imply that LeuO is an important regulator of surface motility.

### LeuO Contributes to *A. baumannii* Adherence and Invasion Onto A549 Cell Line

**Figure 6:**
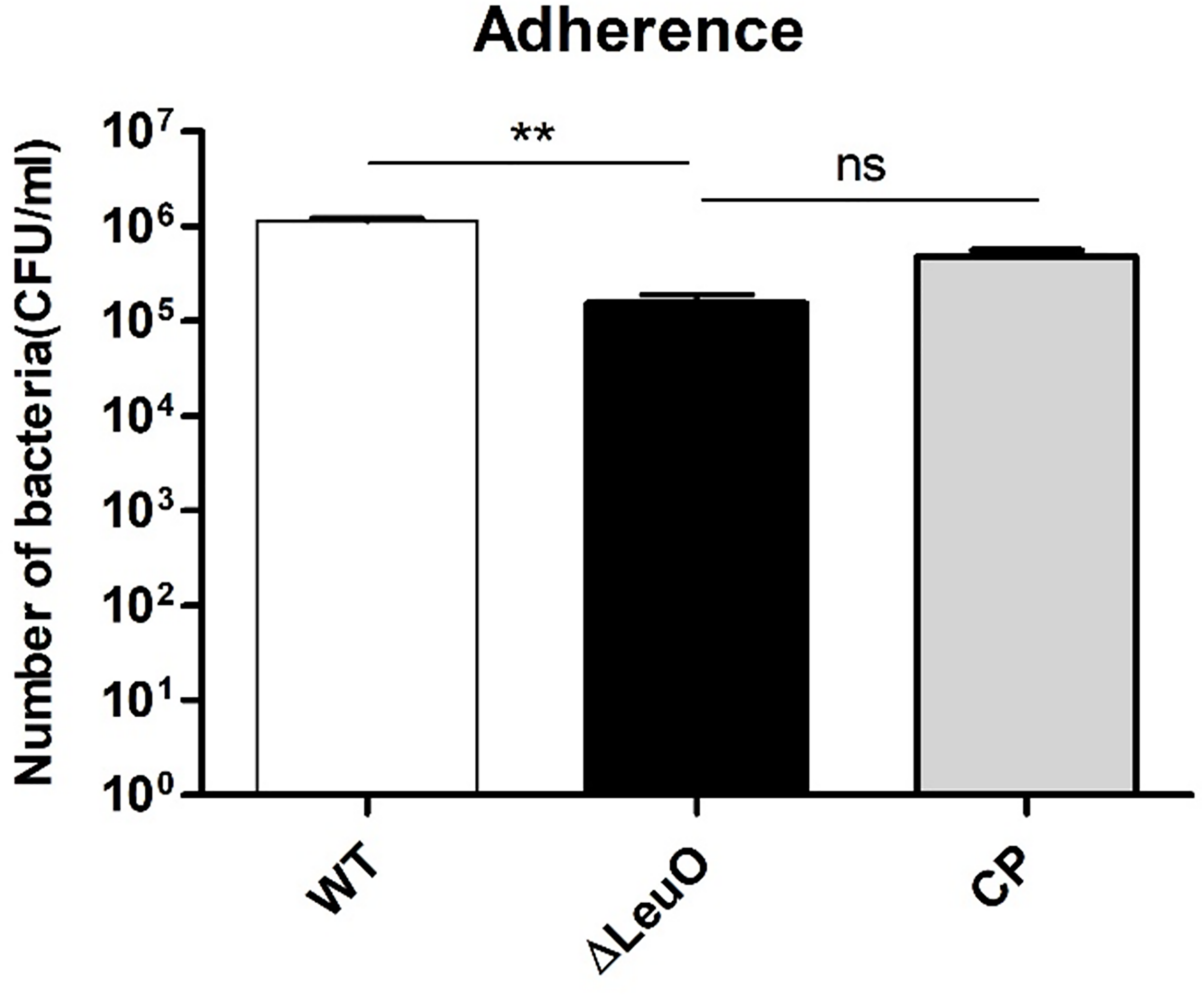
Adherence of *A. baumannii* strains to A549 cells. A549 alveolar epithelial cells were infected with *A. baumannii* wild-type, ΔLeuO, .and CP strains at a MOI of 1:100 for 2 h. After lysis with Triton-X, cell lysates were diluted and plated onto LB agar plates for CFU counting. The experiment was performed with three replicates. Data are presented as mean ± SD. ns, non-significant; **P < 0.01, significantly lower than that of the wild-type strain.

*A. baumannii* pathogenesis largely depends on cellular adhesion and invasion. For determining the importance of LeuO in adherence to human alveolar epithelial cells, we performed an adherence assay on A549 alveolar epithelial cells using *A. baumanni* ATCC 17978 wild-type strain, ΔLeuO strain, and its complementation strain. In contrast to the result of biofilm formation assay on the polystyrene surface, the ΔLeuO strain exhibited approximately 10-fold reduction in attachment compared with that in the wild-type strain (**Figure 6**). This difference in adherence to epithelial cells was statistically significant. The complementation strain exhibited partial recovery of adhesion properties similar to the wild-type strain.

### LeuO Regulates Virulence in Murine Infection

**Figure 7:**
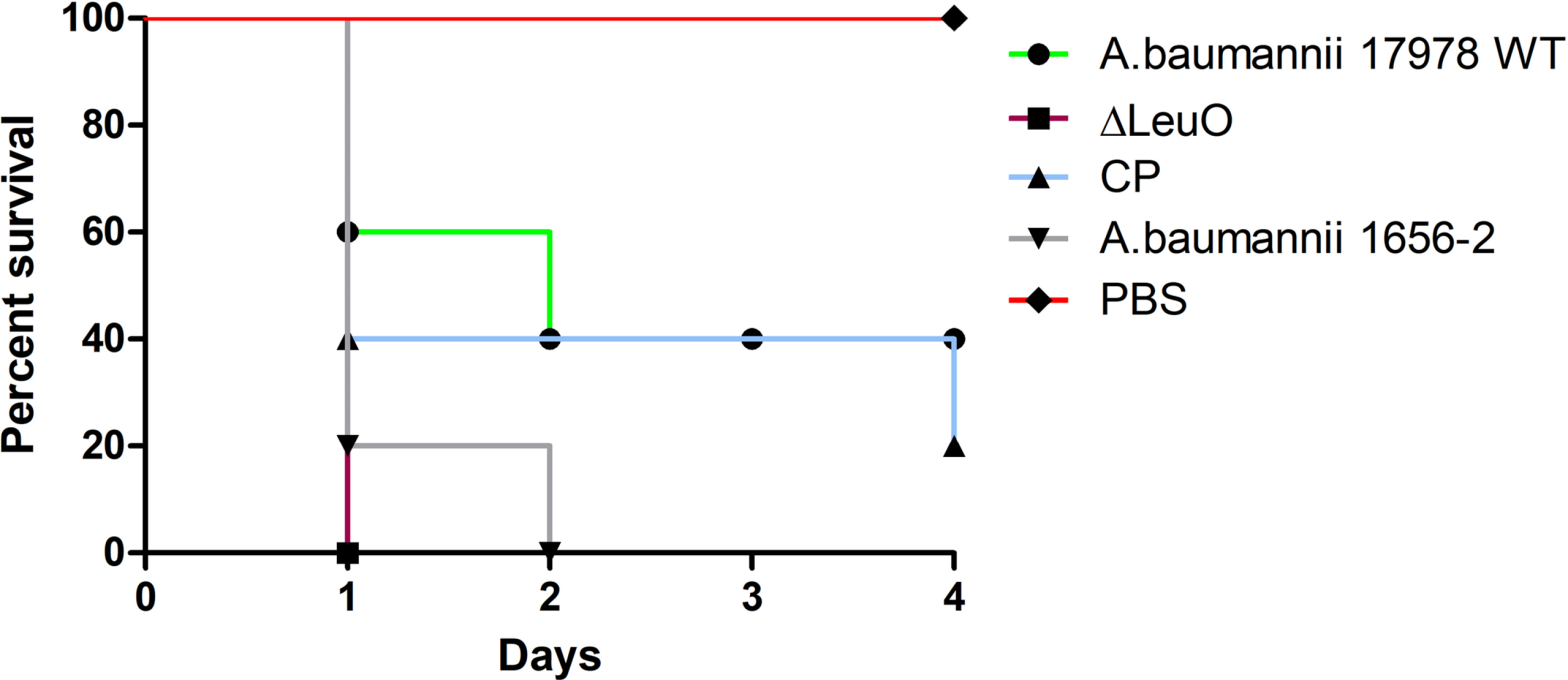
Survival of mice infected with *A. baumannii* strains. Kaplan-Meier survival curves of the virulence of *A. baumannii* 17978 wild-type, ΔLeuO, CP strain and *A. baumannii* 1656-2 in mice were determined up to 4 days of infection. 8-week-old female BALB/c mice were injected intraperitoneally (IP) with 1 × 10^8^ CFU/ml bacterial suspension of tested strains (n = 5 per group). PBS was administered as control group and survival of infected mice were monitored every 12h for 4 days. Survival curve was generated using GraphPad Prism software.

Because of the differences in some virulence-related traits such as biofilm formation and surface motility, we investigated the *in vivo* virulence of *A. baumannii* WT strain, ΔLeuO strain, and CP strain in a mice infection model. Because *A. baumannii* commonly infected immunocompromised patients, neutropenic mice were infected intraperitonially (10^8^ CFU/mouse) and monitored for 96 h postinfection. After bacterial challenge with the LeuO mutant strain, all of mice succumbed to infection within 18 h (**Figure 7**). However, mice exposed to wild-type strain infection survived at 40% till the end of experimental period and all mice (100%) injected with PBS survived during the experimental period. Complementation of LeuO disruption restored the ability of *A. baumannii* to survive during the experimental period. The highly virulent *A. baumannii* 1656-2 strain (Park et al., 2011) showed almost the same result as that of the LeuO mutant strain. The Kaplan–Meier survival curve of the tested strains revealed statistical significance between the strains. Altogether, these data suggested that LeuO was responsible for the virulence of *A. baumannii* in the mice model.

## Discussion

Despite the increasing prevalence of multidrug-resistant strains, the molecular mechanism underlying *A. baumannii* pathogenesis remains poorly defined. Prokaryotes have diverse transcription factors that regulate gene expression to adjust to new environments, among which the LTTR family is highly conserved in bacteria (Rivera-Gómez et al., 2011). In the present study, the predicted 3D structure of *A. baumannii A1S_1874* showed two domains, a DNA-binding domain and a substrate-binding domain, suggesting that *A1S_1874* belongs to the LTTR family (**Figure 1**). LeuO showed the highly conserved region at the N-terminal region compared with other bacterial LTTR family member protein. We suggested that *A1S_1874* encodes LeuO in *A. baumannii* and a new global transcriptional regulator controlled different gene expression and biological functions. In *S. enterica*, LeuO regulates virulence-related genes (Fernández-Mora et al., 2004). In this study, we observed that LeuO is involved in regulating several phenotypes of *A. baumannii*. Our initial identification of the LeuO mutation indicated that there is also no significant difference in the growth of *A. baumannii* strains after LeuO deletion (**Supplementary Figure S2**). This result is consistent with another finding of LTTR deletion in *Listeria monocytogenes* (Abdelhamed et al., 2020). The RND superfamily efflux pump member AdeABC is responsible for resistance to aminoglycoside antibiotics (Marchand et al., 2004). In this experiment, the LeuO mutant exhibited higher susceptibility to the aminoglycoside antibiotics tobramycin, amikacin and gentamicin and also to the widely used beta-lactam antibiotics imipenem and meropenem (**Supplementary Table S2**).

Our RNA-seq data showed that 194 genes were upregulated and 108 genes were downregulated in the LeuO mutant compared with those in the wild-type strain (**Supplementary Table S1**). Differentially expressed genes were further categorized into COG categories to elucidate the potential roles of LeuO in transcriptomic regulation in *A. baumannii* (**Figure 2A**). Transcriptomic data showed that the *csu* operon genes and the *A1S_0112-A1S_0119* cluster were highly upregulated, which are responsible for biofilm formation. The quorum sensing–related genes *A1S_0109* and *A1S_0111* (*AbaI* and *AbaR*) were also upregulated. We also observed that some putative transcriptional regulators of a different family were also upregulated. This could potentially affect different regulatory networks, and hence, further investigation would help understand the role of LeuO in these regulatory networks. We observed that the type VI secretion system-related genes were downregulated, which may play a role in surface motility. A large number of downregulated genes were hypothetical proteins with unknown functions. Further research is necessary to decipher the role of these genes in the *A. baumannii* genome. Previous studies have shown that the members of LTTRs regulate the expression of numerous genes involved in essential bacterial functions, including virulence, motility, metabolism, cell division, and oxidative stress response (Russell et al., 2004; Heroven and Dersch, 2006; Lu et al., 2007; O’Grady et al., 2011). Our transcriptomic data and observation of these LysR family members indicated almost the same effects of gene expression.

Biofilm formation by bacteria provides persistence and protects them from unfavorable conditions, including antimicrobial activity, and it is a major virulence factor (Costerton et al., 1999). A variety of transcriptional regulators have been identified to control biofilm formation in different organisms, for example, the transcriptional regulator LrhA in *E. coli*, CytR in *V. cholerae*, Fur in *Yersinia pestis*, SinR in *Bacillus subtilis*, and LcrX in *Xanthomonas axonopodis* (Haugo and Watnick, 2002; Blumer et al., 2005; Kearns et al., 2005; Sun et al., 2012; Park et al., 2020). LeuO, a putative LTTR, was identified as a repressor of biofilm synthesis in *A. baumannii* for the first time in this study. Our results demonstrated that biofilm formation on abiotic surface was significantly increased in the LeuO mutant strain compared with that in the wild-type strain (**Figure 3**). The *csuA/BABCDE* chaperon-usher secretion system-mediated pili play an important role in biofilm formation (Gaddy and Actis, 2009). Our RNA transcriptomic data revealed that *csu* operon genes were highly upregulated in the LeuO mutant strain. Another gene cluster, *A1S_0112-A1S_0119*, was also highly upregulated. A previous study showed that the *A1S_0112-A1S_0119* cluster is critical for biofilm synthesis (Rumbo-Feal et al., 2017). These findings may explain the cause of high biofilm synthesis in the LeuO mutant strain. LeuO may be a transcriptional regulator that directly or indirectly controls genes that regulate biofilm formation. In contrast, several *A. baumannii* clinical isolates (ST-208, ST-229, ST-357, ST-451, ST-552, and ST-784) exhibited robust biofilm formation, which was almost similar to that of the LeuO mutant strain (**Figure 4A**). Sequence analysis of LeuO locus revealed several amino acid mutations in all the tested clinical strains compared with those in the wild-type strain in the linker helix and regulatory domain regions (**Figure 4B**). These mutations in amino acids may alter LeuO regulation in clinical strains and result in hyper-biofilm formation. It has been reported that mutation in the amino acids of the *B. subtilis* master biofilm regulator *sinR* modulated biofilm formation (Leiman et al., 2014).

*A. baumannii* displays surface-associated motility, which is an important virulence factor (Tomaras et al., 2003). We observed that the surface motility of the LeuO mutant was reduced compared with that in the wild-type strain (**Figure 5**). Extension and retraction of type IV pilus is required for motility (Clemmer et al., 2011). In our RNA-seq data, we observed that the type IV pilus genes *A1S_0646* (*IcmB*) and VGR-like protein (type VI secretion system) *A1S_1288* were downregulated by log 8.29- and 8.3-fold, respectively (**Supplementary Table S1**). Numerous transcriptional factors are known to be involved in the motility of other bacteria, such as ArcB, QseD, and RvoM (Heroven and Dersch, 2006; Habdas et al., 2010; Zhang et al., 2017). These results suggest that LeuO downregulates type IV and type VI pili genes and attenuates motility in the LeuO mutant strain. Surface motility is also controlled by quorum sensing in *A. baumannii.* Although abaRI genes were upregulated in LeuO mutant strain, surface motility was reduced. Further research will help to describe this discrepancy.

In the present study, we used alveolar epithelial cells (A549) to determine the role of LeuO in *A. baumannii* adherence. The LeuO mutant displayed significantly lower adherence to A549 epithelial cells than that shown by the wild-type strain (**Figure 6**). Our observations of the LeuO-mediated epithelial cell adherence and biofilm formation on abiotic surface are quite opposite, which suggests the presence of different mechanisms of adherence to either biotic or abiotic surface. Although *csu* operon is essential for biofilm formation, other studies have suggested that *A. baumannii* adherence to abiotic surfaces is independent of *csu*A/BABCDE-mediated pili and that *csu*-knockout strains showed no difference in binding to bronchial cells (de Breij et al., 2009; Gaddy and Actis, 2009). Our data corroborate with these findings. Further research is necessary to understand the exact molecular mechanisms involved in adherence to epithelial cells.

Several LTTRs are known to control the expression of virulence genes to maintain the host–pathogen interaction, such as ShvR in *B. cenocepacia*, MexT in *P. aeruginosa*, and LeuO in *S. enterica* serovar *Typhimurium* (Tropel and van der Meer, 2004; Tian et al., 2009; Espinosa and Casadesús, 2014). In this study, we observed that LeuO regulated several genes related to virulence. In our study, LeuO deletion increased lethal infection in the intraperitoneal mouse infection model (**Figure 7**). This increase in pathogenicity may be due to the result of increased virulence-related gene expression. Some previous studies have shown that disruption of LTTR family members such as GigC, MvfR, and ShvR reduced virulence in mammalian and plant models of infection (Cao et al., 2001; Déziel et al., 2005; Gebhardt et al., 2020). This contrasting result requires further analysis to understand the actual role of LTTRs in *A. baumannii* pathogenicity.

In conclusion, LeuO (*A1S_1874*) was identified as an LTTR, and transcriptome analysis revealed that LeuO regulated divergent sets of genes with different biological functions that were altered after LeuO deletion. Altogether, LeuO is involved in the regulation of biofilm formation, adherence, motility, and virulence of *A. baumannii*. This study provides valuable information regarding the role of an LTTR in the pathogenesis of *A. baumannii.*

### Statistical Analysis

All experiments in this study were performed independently, and data are expressed as mean and standard deviations (SDs). All raw data were saved in Excel files and imported to GraphPad Prism for statistical analysis. Statistical differences between groups of data were compared using Student’s *t*-tests or one-way analysis of variance along with Turkey’s multiple comparisons.

## Data Availability Statement

The findings of this study are available within this paper and its **Supplementary Material**. The transcriptomic data discussed in this publication have been deposited in the NCBI’s gene expression omnibus database (http://www.ncbi.nlm.nih.gov/geo/) and can be accessed using the accession number GSE173626.

## Ethics Statement

Animal experiments were conducted according to experimental procedures approved by the Animal Care Committee of Kyungpook National University, South Korea (approval number: KNU-2019-178).

## Author Contributions

MI, KK, and MS designed the study and MI wrote the manuscript. MI, KK, and MS performed the experiments. JL reviewed the manuscript. All authors contributed to the article and approved the submitted version.

## Funding

This study was supported by a grant from the Korea Government National Research Foundation Grants 2016R1D1A1B01008960 (to MS).

## Conflict of Interest

The authors declare that the research was conducted in the absence of any commercial or financial relationships that could be construed as a potential conflict of interest.

## Publisher’s Note

All claims expressed in this article are solely those of the authors and do not necessarily represent those of their affiliated organizations, or those of the publisher, the editors and the reviewers. Any product that may be evaluated in this article, or claim that may be made by its manufacturer, is not guaranteed or endorsed by the publisher.
