# Supplementary material for: LeuO, a LysR-Type Transcriptional Regulator, Is Involved in Biofilm Formation and Virulence of Acinetobacter baumannii
Source: Front Cell Infect Microbiol. 2021 Oct 11;11:738706. doi: 10.3389/fcimb.2021.738706 (PMC8543017; doi:10.3389/fcimb.2021.738706)
Supplement: Supplementary file 1 [file Table_1.docx]

Supplementary Material

# Supplementary Data


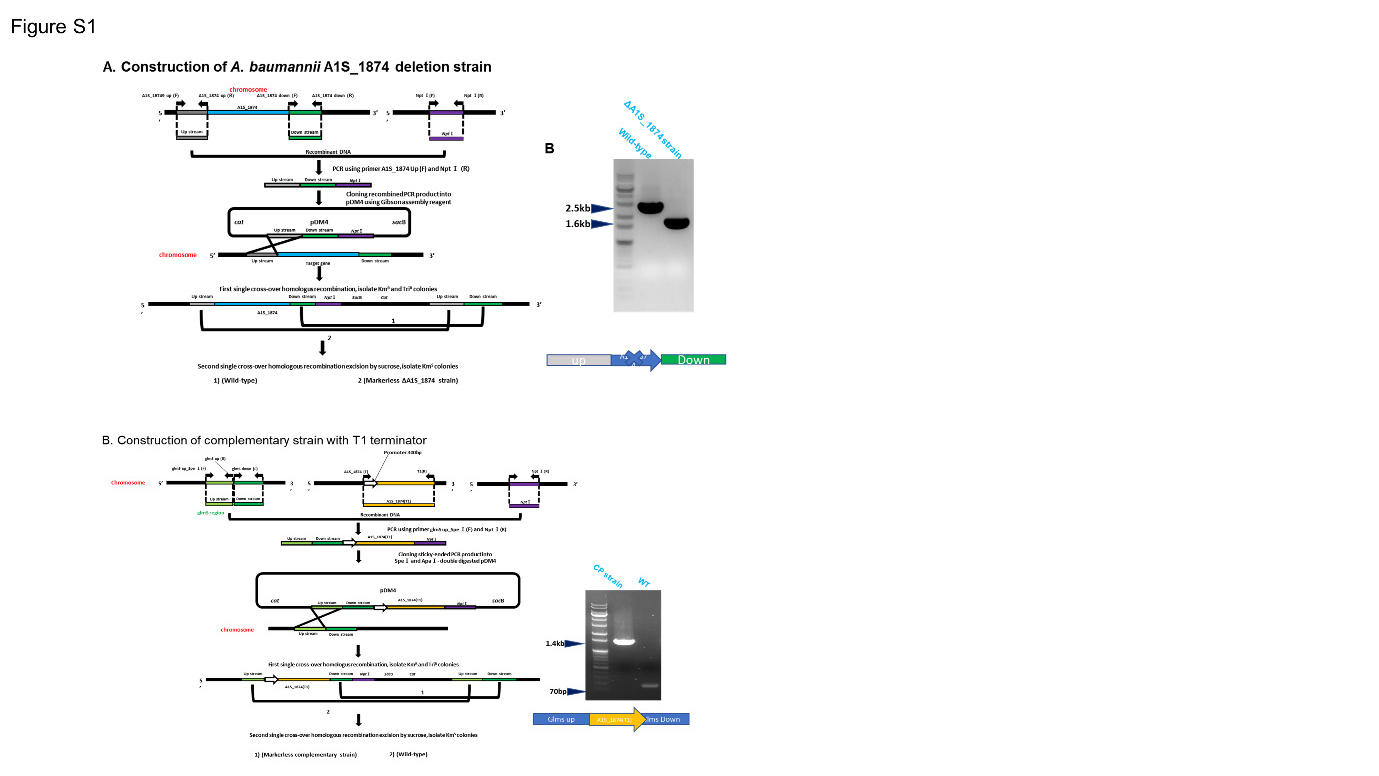


**Supplementary Figure 1**. Schematic representation of marker-less *A1S_1874* deletion and *A1S_1874* complementation constructed using homologous recombination techniques. **(A)** *A. baumannii* *A1S_1874* upstream and downstream regions and *npt1* (conferring resistance to kanamycin) were amplified. Combined DNA fragments were cloned into PDM4 and integrated into chromosome by conjugation and homologous recombination. Excision of plasmid region with *npt1* from chromosome was achieved using sucrose. The deletion mutant was confirmed by PCR. *sacB* levansucrose-encoding gene, *cat* chloramphenicol-resistant gene, *Kan^R^* kanamycin-resistant. Right panel shows the PCR analysis results of wild-type (WT) and A1S-1874 mutant (∆A1S_1874) strains using 1% agarose gel. **(B)** *A. baumannii* *A1S_1874* with its 300-bp promoter region, T1 terminator region, and *npt1* (conferring resistance to kanamycin) were amplified. Combined DNA fragments were cloned into PDM4 and integrated into chromosome by conjugation and homologous recombination. Right panel shows the PCR analysis results of wild-type (WT) and A1S-1874 complementary (CP) strains using 1% agarose gel.


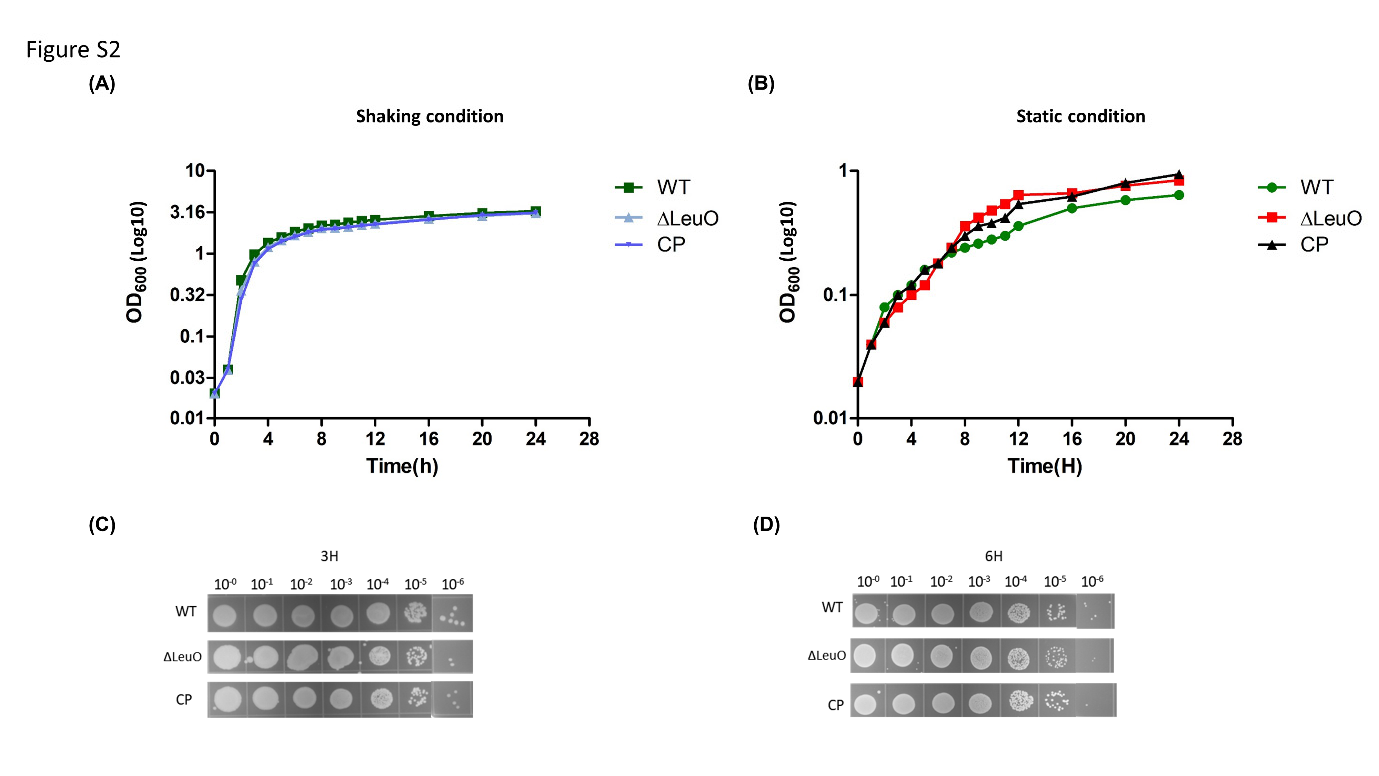


**Supplementary Figure S2.** LeuO gene deletion has no impact on *A. baumannii* growth

**(A, B)** Growth assay was conducted with *A. baumannii* 17978 WT, ∆LeuO, and complementary strains in LB broth under shaking and static conditions. Growth pattern was determined by measuring the optical density at 600 nm for 24 h. Three independent experiments were performed to collect growth data. **(C, D)** CFUs of *A. baumannii* strains were counted after culturing the sample for 3 h under shaking condition and for 6 h under static condition. Samples were serially diluted, and 20 µL of sample was plated onto LB agar plates and placed in a 37°C incubator for 17 h. CFUs were determined, and plates were photographed.


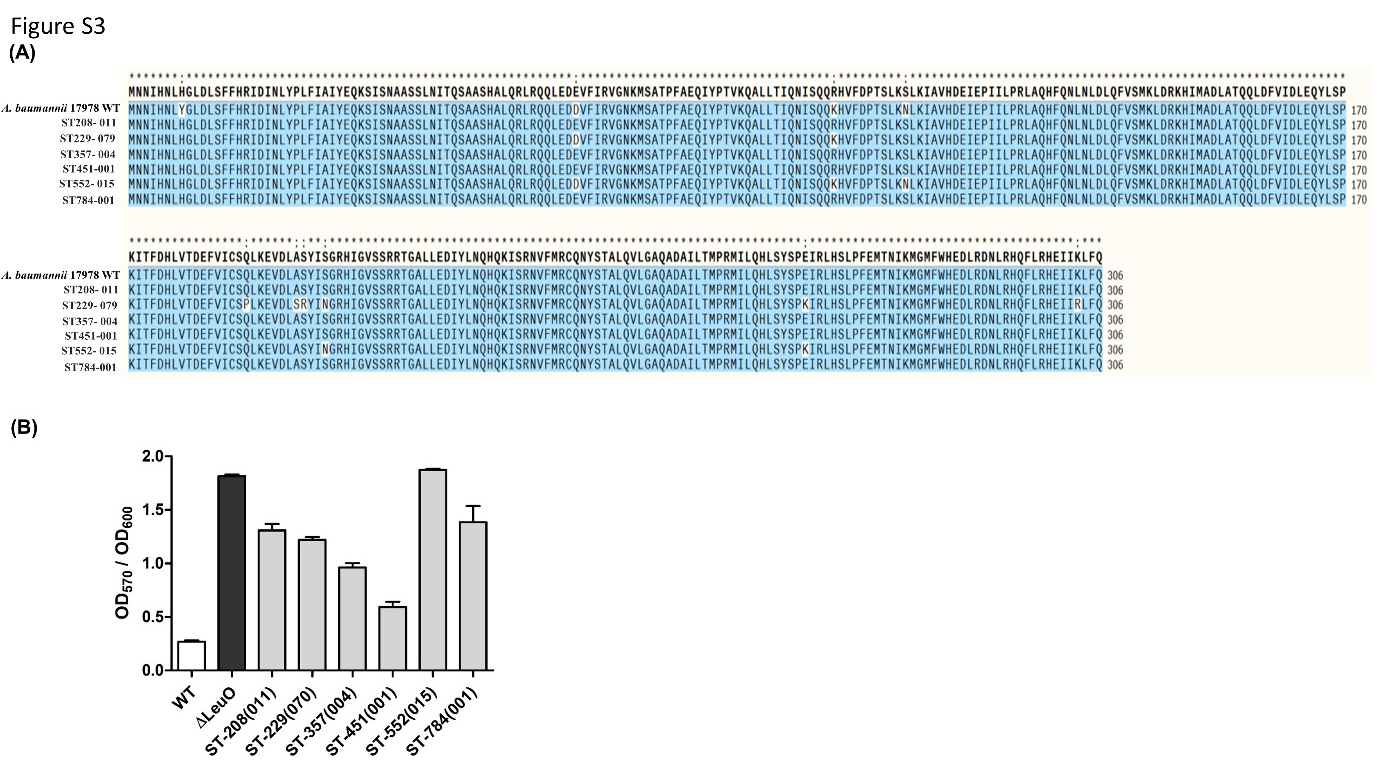
**Supplementary Figure S3.** Hyper-biofilm-forming clinical strains have point mutation in LeuO gene locus

**(A)** Multiple sequence alignment of *A. baumannii* 17978 *A1S_1874* with clinical *A. baumannii* strains ST-208(011), ST-229(079), ST-357(004), ST-451(001), ST-552(015), and ST-784(001) using COBALT: Multiple Alignment Tool. “*”, “:” indicate most conserved residues and semi-conserved sequence, respectively. Point mutation is shown in the white color background. **(B)** Biofilm values (OD_570_) of *A. baumannii* 17978 wild-type, ∆LeuO, and clinical strains were normalized by growth levels (OD_600_) to compensate for the levels of biofilm formation on polystyrene surface. Biofilm formation assays were conducted in triplicate, and average values of three replicates were plotted with standard deviation.


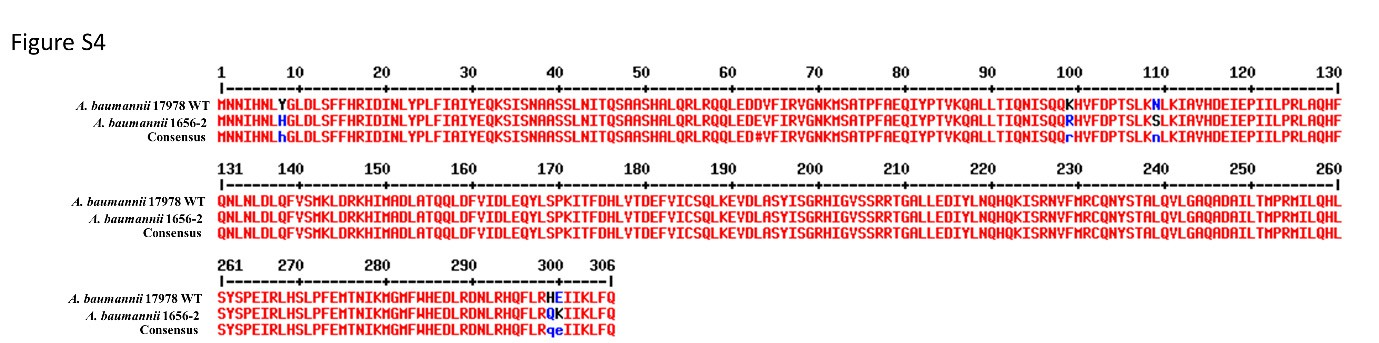


**Supplementary Figure S4.** Alignment of *A. baumannii* 17978 *A1S_1874* and *A. baumannii* 1656-2

Amino acid sequence alignment of *A. baumannii* 17978 *A1S_1874* with clinical *A. baumannii* strains 1656-2 using Multiple Alignment Tool. Point mutation is shown in the blue color.

**Methods**

**Construction of ∆A1S_1874 mutant strain**

*A. baumannii* A1S-1874 gene deletion mutant was constructed using the marker-less gene deletion method as described by Jung et al^1^. The NCBI database was used to collect the DNA sequence with the open reading frame (ORF) of *A. baumannii* ATCC 17978 *A1S_1874*. The homologous recombination technique was used to design the primer sets that led to the overlap of the sequence (842 bp) upstream of A1S_1874 and (746 bp) downstream of A1S_1874, and for antibiotic selection (KnR), the NptI fragment (1233 bp) was used. *A. baumannii* ATCC 17978 was used to amplify the upstream and downstream fragments, and pOH4 ^2^ plasmid was used for NptI. The primers used in this experiment are listed in Supplementary Table S4. Crossover PCR was performed to obtain the complete insert DNA strand. Recombined full-length insert and pDM4 were ligated using Gibson Assembly Master Mix (BioLabs), and after obtaining the correct cloned colony, it was confirmed by sequencing analysis. Plasmid pDM4:A1S_1874 was transformed into the *E. coli* sm10 λ pir strain for conjugation. *E. coli* sm10 λ pir cells containing the insert acted as donor cells to *A. baumannii* ATCC 17978 cells (recipient cells) during the overnight incubation of the bacterial mixture on a fresh LB agar plate at 30°C. Tri/Kan LB agar plate was used for first selection, and 10% (wt/vol) sucrose LB agar plate was used for second selection to identify the *A. baumannii* strain that had correctly undergone homologous recombination.

**Construction of complementary (CP) strain**

For *A1S_1874* complementation in *A. baumannii* Δ*A1S_1874* mutant, *A1S_1874*, including its native promoter region, was inserted into the *glmS* region of an *A. baumannii* *A1S_1874*-deficient strain using overlap extension PCR as described earlier. *A1S_1874* region with its native promoter and T1 terminator, *glmS* upstream region, *glmS* downstream region, and kanamycin-resistant cassette (NptI*)* were amplified using primers listed in Table S4. Primer sets *glmS* up F and *Npt1* were used for overlap extension PCR of four PCR products. The combined DNA fragment produced by the overlap extension PCR was digested with SpeI (Enzynomics Inc., Korea) and ApaI (Enzynomics Inc., Korea). The digested insert and pDM4 plasmid were ligated to generate *A1S_1874* deletion and complementary strains (Table S3). The chimeric plasmid was integrated into the chromosome of *A*. *baumannii* Δ*A1S_1874* mutant using the homologous recombination method. The cloned product was confirmed by DNA sequencing.

**Bacterial growth pattern and colony-forming units (CFUs)**

The growth rate of each bacterial strain *(A. baumannii* ATCC 17978, ∆LeuO, and CP) was determined by inoculating in LB broth. Overnight cultures of bacterial strains were adjusted to a value of OD_600_ = 1.0 and diluted at a ratio of 1:100 using fresh LB. Freshly inoculated culture was incubated at 37°C for 24 h under shaking and static conditions. Bacterial growth was determined every 1 h at OD_600_ using a spectrophotometer (Biochrom WPA CO8000 Cell Density Meter). This experiment was performed at least three times.

The CFU values of each strain (*A. baumannii* ATCC 17978, ∆LeuO, and CP) were determined after culturing in LB broth. First, samples were collected in a time-dependent manner and then 10-fold serially diluted with PBS. A volume of 20 μL of the diluted sample was placed on each designated LB agar plate. The plates were then dried for 1 h and placed in a 37°C incubator for 17 h. CFUs were determined, and plates were photographed. This assay was performed in duplicate.

**Antimicrobial susceptibility testing**

The minimum inhibitory concentrations (MICs) of the following antimicrobial agents were determined using the broth dilution method according to CLSI guidelines (CLSI, 2015): ceftazidime, meropenem, imipenem, aztreonam, amikacin, gentamycin, tobramycin, tetracycline, trimethoprim, colistin, ciprofloxacin, and levofloxacin. A 0.5-McFarland standard bacterial suspension was prepared from overnight bacterial cultures and diluted with modified MHB broth (10-fold dilution). A 96-well plate was used to prepare different concentrations of antibiotics and incubated at 37°C for 18 h. The MICs of each antibiotic were obtained within the expected range of *E. coli* ATCC 25922 and *P. aeruginosa* ATCC 27853 as quality control strains according to Clinical Laboratory Standards Institute (CLSI, 2015). The antimicrobial susceptibility of *A. baumanni*i WT, ∆LeuO, and CP strains was evaluated in triplicate, and the MIC value of each strain was calculated.

**Table S1.A. Up-regulated genes in the *A. baumannii* 17978 ΔLeuO compared to *A. baumannii* 17978 WT.**

Table S1.A. Up-regulated genes in the *A. baumannii* 17978 ΔLeuO compared to *A. baumannii* 17978 WT.

| **Locus tag** | **Gene product** | **Fold change (∆LeuO/WT)** |
| --- | --- | --- |
| A1S_0114 | acyl carrier protein | **201.609303** |
| A1S_0113 | acyl-CoA dehydrogenase | **182.924329** |
| A1S_0112 | acyl-CoA synthetase/AMP-acid ligases II | **153.131306** |
| A1S_1256 | transcriptional regulator | **150.432283** |
| A1S_0115 | amino acid adenylation | **139.667343** |
| A1S_0116 | RND superfamily transporter | **102.401645** |
| A1S_2218 | protein CsuA/B | **64.168028** |
| A1S_0628 | transposase | **60.313515** |
| A1S_1292 | signal peptide | **43.036006** |
| A1S_0745 | hypothetical protein | **31.715922** |
| A1S_1294 | hypothetical protein | **29.282490** |
| A1S_0117 | hypothetical protein | **23.706489** |
| A1S_1383 | surface antigen | **21.161435** |
| A1S_0109 | homoserine lactone synthase | **20.734331** |
| A1S_2216 | protein CsuB | **17.846972** |
| A1S_2347 | hypothetical protein | **16.969383** |
| A1S_1385 | hypothetical protein | **16.250592** |
| A1S_1357 | alanine racemase | **15.072312** |
| A1S_2215 | protein CsuC | **14.043668** |
| A1S_2214 | protein CsuD | **13.456508** |
| A1S_0118 | hypothetical protein | **13.051833** |
| A1S_1386 | catalase | **12.280301** |
| A1S_1295 | hypothetical protein | **11.490974** |
| A1S_2213 | protein CsuE | **10.958343** |
| A1S_2217 | protein CsuA | **10.400471** |
| A1S_1384 | CinA-like protein | **10.041875** |
| A1S_0119 | phosphopantethiene-protein transferase | **9.511892** |
| A1S_0804 | trehalose-6-phosphate phophatase | **7.404987** |
| A1S_0110 | hypothetical protein | **7.199053** |
| A1S_1438 | coenzyme F420-dependent N5N10-methylene tetrahydromethanopterin reductase | **7.047579** |
| A1S_1408 | rhodanese-related sulfurtransferase | **7.010835** |
| A1S_0803 | trehalose-6-phosphate synthase | **6.756441** |
| A1S_1439 | coenzyme F420-dependent N5N10-methylene tetrahydromethanopterin reductase | **6.710649** |
| A1S_0980 | ferric enterobactin receptor precursor | **6.128518** |
| A1S_1407 | serine acetyltransferase | **6.050651** |
| A1S_0452 | hypothetical protein | **5.570219** |
| A1S_2074 | hypothetical protein | **5.427516** |
| A1S_1406 | major membrane protein I (MMP-I) | **5.383128** |
| A1S_0741 | hypothetical protein | **5.036271** |
| A1S_0549 | hypothetical protein | **4.832236** |
| A1S_0242 | ferrous iron transport protein A | **4.743282** |
| A1S_2649 | regulatory protein | **4.702285** |
| A1S_2230 | hypothetical protein | **4.697201** |
| A1S_3174 | regulatory or redox component complexing with Bfr in iron storage and mobility (BFD) | **4.653110** |
| A1S_1587 | EsvK2 | **4.633678** |
| A1S_0971 | B12-dependent methionine synthase | **4.454880** |
| A1S_2648 | hypothetical protein | **4.434055** |
| A1S_1509 | pili assembly chaperone | **4.414595** |
| A1S_2676 | tRNA-Leu | **4.347946** |
| A1S_0243 | ferrous iron transport protein B | **4.124860** |
| A1S_0742 | iron-regulated protein | **4.047042** |
| A1S_1366 | amino acid transporter LysE | **4.004486** |
| A1S_2654 | periplasmic binding protein of transport/transglycosylase | **3.921338** |
| A1S_1291 | hypothetical protein | **3.894551** |
| A1S_1510 | fimbrial protein | **3.885610** |
| A1S_1405 | cysteine desulfurase 1 (Csd) | **3.869296** |
| A1S_1440 | MFS family transporter | **3.814800** |
| A1S_1079 | dichlorophenol hydroxylase | **3.795227** |
| A1S_1466 | glutaminase-asparaginase | **3.723174** |
| A1S_1063 | TonB-dependent siderophore receptor | **3.707930** |
| A1S_2229 | acyl-CoA dehydrogenase-related protein | **3.684571** |
| A1S_1588 | Phage terminase-like protein large subunit | **3.599666** |
| A1S_1586 | EsvK1 | **3.593588** |
| A1S_3184 | tRNA-Glu | **3.587302** |
| A1S_0717 | tRNA-Pro | **3.493894** |
| A1S_0805 | hydrolase biotin biosynthesis (BioH) | **3.446534** |
| A1S_2038 | hypothetical protein | **3.373294** |
| A1S_0081 | signal peptide | **3.372605** |
| A1S_2559 | hypothetical protein | **3.357588** |
| A1S_1591 | phage major capsid protein HK97 | **3.350527** |
| A1S_1773 | RND family drug transporter | **3.348775** |
| A1S_1403 | cysteine desulfurase 1 (Csd) | **3.339552** |
| A1S_3188 | tRNA-Glu | **3.319768** |
| A1S_2988 | hypothetical protein | **3.299120** |
| A1S_1698 | lipoyl synthase | **3.226898** |
| A1S_0453 | biopolymer transport protein (ExbB) | **3.201660** |
| A1S_1078 | hypothetical protein | **3.138238** |
| A1S_1648 | lysine/ornithine N-monooxygenase | **3.109250** |
| A1S_0981 | ferric enterobactin receptor precursor | **3.094955** |
| A1S_2798 | hypothetical protein | **3.085833** |
| A1S_1772 | MFS family transporter | **3.083856** |
| A1S_1703 | dihydrolipoamide dehydrogenase | **3.053149** |
| A1S_0636 | DNA polymerase V component | **3.016138** |
| A1S_2278 | alpha/beta family hydrolase | **3.007616** |
| A1S_2553 | transposition site target selection protein D | **2.999881** |
| A1S_0111 | transcriptional regulator | **2.999374** |
| A1S_2513 | tRNA-Asn | **2.971452** |
| A1S_3339 | ferric siderophore receptor protein | **2.970225** |
| A1S_3019 | tRNA-Arg | **2.949282** |
| A1S_2228 | hypothetical protein | **2.946563** |
| A1S_1064 | tRNA-Glu | **2.944568** |
| A1S_1594 | hypothetical protein | **2.934786** |
| A1S_1699 | acetoin:26-dichlorophenolindophenol oxidoreductase subunit alpha | **2.929779** |
| A1S_2041 | hypothetical protein | **2.904520** |
| A1S_2509 | chaperone | **2.883708** |
| A1S_0689 | p-aminobenzoate synthetase | **2.859642** |
| A1S_2039 | hypothetical protein | **2.856428** |
| A1S_2042 | TetR family transcriptional regulator | **2.850074** |
| A1S_0244 | hypothetical protein | **2.840773** |
| A1S_1530 | SSS family major sodium/proline symporter | **2.826592** |
| A1S_1702 | dihydrolipoamide dehydrogenase | **2.819757** |
| A1S_1508 | fimbrial biogenesis outer membrane usher protein | **2.795607** |
| A1S_2382 | BasD | **2.794265** |
| A1S_0072 | GntR family transcriptional regulator | **2.788964** |
| A1S_1736 | hypothetical protein | **2.766937** |
| A1S_1090 | AsnC family transcriptional regulator | **2.748959** |
| A1S_0806 | adenosylmethionine-8-amino-7-oxononanoate aminotransferase | **2.732750** |
| A1S_2008 | DNA repair protein | **2.724442** |
| A1S_2345 | tRNA-Asn | **2.692308** |
| A1S_1499 | hypothetical protein | **2.684461** |
| A1S_2024 | glutamate 5-kinase | **2.677646** |
| A1S_1467 | glutamate symport transmembrane protein | **2.614469** |
| A1S_0548 | TetR family transcriptional regulator | **2.612608** |
| A1S_3273 | peptide signal | **2.606973** |
| A1S_0809 | dethiobiotin synthetase x | **2.601751** |
| A1S_1223 | tRNA-Met | **2.587753** |
| A1S_2226 | glycosyl transferase related protein | **2.553407** |
| A1S_3146 | multidrug ABC transporter | **2.551679** |
| A1S_0073 | 2-methylisocitrate lyase | **2.517072** |
| A1S_1647 | siderophore biosynthesis protein | **2.516160** |
| A1S_1033 | hypothetical protein | **2.509495** |
| A1S_1700 | acetoin:26-dichlorophenolindophenol oxidoreductase subunit beta | **2.505733** |
| A1S_2026 | hypothetical protein | **2.505343** |
| A1S_1224 | transposase | **2.486394** |
| A1S_2021 | hypothetical protein | **2.481105** |
| A1S_0831 | tRNA-Gln | **2.460286** |
| A1S_0692 | protein FilC | **2.459069** |
| A1S_2025 | hypothetical protein | **2.431545** |
| A1S_1504 | purine-cytosine permease | **2.420540** |
| A1S_2567 | thioesterase | **2.410910** |
| A1S_2080 | siderophore receptor | **2.403863** |
| A1S_1590 | peptidase U35 phage prohead HK97 | **2.397078** |
| A1S_2027 | hypothetical protein | **2.396108** |
| A1S_0807 | 8-amino-7-oxononanoate synthase | **2.392262** |
| A1S_2031 | hypothetical protein | **2.381688** |
| A1S_0693 | protein FilD | **2.373383** |
| A1S_0808 | biotin biosynthesis protein (BioC) | **2.369981** |
| A1S_1497 | acyltransferase | **2.368692** |
| A1S_2125 | VIC family potassium channel protein | **2.363992** |
| A1S_2030 | phage associated protein | **2.359972** |
| A1S_1592 | Phage head-tail adaptor | **2.357466** |
| A1S_0719 | zinc-binding dehydrogenase | **2.355180** |
| A1S_1507 | fimbrial protein | **2.354095** |
| A1S_1701 | dihydrolipoamide acetyltransferase | **2.344851** |
| A1S_0277 | tRNA-Gly | **2.341057** |
| A1S_1649 | RND efflux transporter | **2.332625** |
| A1S_1387 | oxidoreductase | **2.310538** |
| A1S_2909 | tRNA-Leu | **2.309013** |
| A1S_2029 | hypothetical protein | **2.308651** |
| A1S_1595 | hypothetical protein | **2.304236** |
| A1S_1296 | hypothetical protein | **2.297784** |
| A1S_0657 | transposase | **2.291317** |
| A1S_2803 | tRNA-Asp | **2.283618** |
| A1S_0120 | tRNA-Gly | **2.282960** |
| A1S_1158 | signal peptide | **2.279106** |
| A1S_1737 | 3-hydroxybutyrate dehydrogenase | **2.275494** |
| A1S_1596 | hypothetical protein | **2.267690** |
| A1S_0454 | biopolymer transport protein (ExbD) | **2.262460** |
| A1S_3043 | hypothetical protein | **2.257527** |
| A1S_0832 | tRNA-Gln | **2.245504** |
| A1S_1404 | cysteine desulfurase 1 (Csd) | **2.243298** |
| A1S_1657 | siderophore biosynthesis protein | **2.232585** |
| A1S_1498 | TetR family transcriptional regulator | **2.230366** |
| A1S_0016 | site-specific tyrosine recombinase | **2.222298** |
| A1S_1032 | hypothetical protein | **2.200786** |
| A1S_1583 | hypothetical protein | **2.199948** |
| A1S_0664 | replication C family protein | **2.178136** |
| A1S_2022 | tail fiber | **2.173980** |
| A1S_0846 | tRNA-Met | **2.173286** |
| A1S_1162 | hypothetical protein | **2.168743** |
| A1S_2386 | ferric acinetobactin binding protein | **2.167072** |
| A1S_0711 | methylated-DNA-(protein)-cysteine S- methyltransferase | **2.165048** |
| A1S_0401 | tRNA-Lys | **2.158854** |
| A1S_3216 | tRNA-Phe | **2.147043** |
| A1S_0474 | ferric siderophore receptor protein | **2.134895** |
| A1S_2016 | phage-related lysozyme | **2.134676** |
| A1S_1173 | RumB | **2.132952** |
| A1S_2081 | TonB-dependent siderophore receptor | **2.129645** |
| A1S_1388 | hypothetical protein | **2.129246** |
| A1S_1593 | hypothetical protein | **2.127995** |
| A1S_2536 | ATPase | **2.112165** |
| A1S_2017 | hypothetical protein | **2.079606** |
| A1S_0710 | SMR family drug transporter | **2.075055** |
| A1S_2141 | potassium-transporting ATPase subunit A | **2.070801** |
| A1S_2032 | hypothetical protein | **2.063588** |
| A1S_1778 | methylenetetrahydrofolate reductase | **2.051340** |
| A1S_2537 | LysR-type transcriptional regulator | **2.049871** |
| A1S_2160 | hemin storage system HmsR protein | **2.047035** |
| A1S_2390 | acinetobactin biosynthesis protein | **2.045380** |
| A1S_0830 | tRNA-Gln | **2.038550** |
| A1S_3018 | tRNA-Arg | **2.017231** |
| A1S_1379 | SAM-dependent methyltransferase | **2.016096** |
| A1S_2236 | tRNA-Trp | **2.012886** |
| A1S_2028 | phage putative head morphogenesis protein | **2.003300** |

Table S1.B. Down-regulated genes in the *A. baumannii* 17978 ΔLeuO compared to *A. baumannii* 17978 WT.

| **Locus tag** | **Gene product** | **Fold change (∆LeuO/WT)** |
| --- | --- | --- |
| A1S_0645 | hypothetical protein | **-16.028664** |
| A1S_0644 | hypothetical protein | **-12.477243** |
| A1S_0800 | bacterioferritin | **-8.595236** |
| A1S_1288 | VGR-like protein | **-8.298315** |
| A1S_0646 | IcmB protein | **-8.294988** |
| A1S_0627 | hypothetical protein | **-8.144410** |
| A1S_1227 | amino acid transporter LysE | **-7.471356** |
| A1S_0640 | hypothetical protein | **-7.455214** |
| A1S_2304 | RND efflux transporter | **-5.943429** |
| A1S_2305 | cation/multidrug efflux pump | **-5.557192** |
| A1S_0642 | hypothetical protein | **-5.551966** |
| A1S_0631 | hypothetical protein | **-5.060727** |
| A1S_0630 | hypothetical protein | **-4.945136** |
| A1S_3175 | bacterioferritin | **-4.929128** |
| A1S_0736 | hypothetical protein | **-4.782686** |
| A1S_1266 | hypothetical protein | **-4.514323** |
| A1S_2675 | tRNA-Cys | **-4.479449** |
| A1S_1289 | VGR-like protein | **-4.474505** |
| A1S_2306 | RND efflux transporter | **-4.180660** |
| A1S_3364 | VGR-like protein | **-4.029551** |
| A1S_2452 | NAD-dependent aldehyde dehydrogenases | **-4.003637** |
| A1S_1093 | arginine/ornithine N-succinyltransferase subunit beta | **-3.992374** |
| A1S_0643 | hypothetical protein | **-3.871826** |
| A1S_1268 | hypothetical protein | **-3.846051** |
| A1S_0641 | hypothetical protein | **-3.820614** |
| A1S_1092 | succinylornithine transaminase (carbon starvation protein C) | **-3.770579** |
| A1S_2098 | alcohol dehydrogenase | **-3.755553** |
| A1S_1267 | lactam utilization protein | **-3.727898** |
| A1S_0737 | 5-methyltetrahydropteroyltriglutamate/homocysteine S-methyltransferase | **-3.570771** |
| A1S_0647 | IcmO protein | **-3.534416** |
| A1S_2102 | aldehyde dehydrogenase 1 | **-3.104413** |
| A1S_0268 | DNA binding protein | **-3.093698** |
| A1S_2449 | aromatic amino acid APC transporter | **-3.054639** |
| A1S_1839 | dihydroxy-acid dehydratase | **-3.052461** |
| A1S_1390 | hypothetical protein | **-3.023925** |
| A1S_3251 | amino acid transporter LysE | **-3.017377** |
| A1S_0650 | conjugal transfer protein | **-3.002049** |
| A1S_0648 | hypothetical protein | **-2.944659** |
| A1S_0634 | hypothetical protein | **-2.760918** |
| A1S_1505 | hypothetical protein | **-2.752738** |
| A1S_1342 | enoyl-CoA hydratase | **-2.742690** |
| A1S_3281 | 4-aminobutyrate aminotransferase | **-2.728747** |
| A1S_2450 | pyruvate decarboxylase | **-2.701080** |
| A1S_1503 | transmembrane pair | **-2.699736** |
| A1S_1111 | p-hydroxycinnamoyl CoA hydratase/lyase | **-2.650510** |
| A1S_1075 | D-amino-acid dehydrogenase | **-2.639975** |
| A1S_1270 | hypothetical protein | **-2.597792** |
| A1S_1269 | allophanate hydrolase subunit 1 and 2 | **-2.579865** |
| A1S_0738 | flavoprotein oxidoreductase | **-2.578271** |
| A1S_1091 | succinylornithine transaminase (carbon starvation protein C) | **-2.552536** |
| A1S_3426 | formate/nitrate transporter | **-2.534003** |
| A1S_2401 | hypothetical protein | **-2.525521** |
| A1S_1835 | aldehyde dehydrogenase | **-2.466184** |
| A1S_1836 | hypothetical protein | **-2.419846** |
| A1S_2006 | response regulator protein | **-2.418492** |
| A1S_0629 | hypothetical protein | **-2.415012** |
| A1S_1760 | hypothetical protein | **-2.412968** |
| A1S_3248 | glycerol uptake facilitator | **-2.404187** |
| A1S_0030 | alkanesulfonate transport protein | **-2.378114** |
| A1S_0624 | lipoprotein | **-2.354798** |
| A1S_3047 | oligopeptidase A | **-2.344493** |
| A1S_1488 | Acyl-CoA dehydrogenase | **-2.344440** |
| A1S_2336 | hypothetical protein | **-2.343927** |
| A1S_1417 | amino-acid acetyltransferase | **-2.323888** |
| A1S_1121 | lipase/esterase | **-2.322256** |
| A1S_0632 | DNA primase | **-2.306380** |
| A1S_1089 | hypothetical protein | **-2.285344** |
| A1S_1412 | glutathione S-transferase-like protein | **-2.266715** |
| A1S_1758 | short-chain dehydrogenase | **-2.259295** |
| A1S_0550 | VGR-like protein | **-2.257698** |
| A1S_1077 | hypothetical protein | **-2.237423** |
| A1S_0955 | short-chain dehydrogenase | **-2.235947** |
| A1S_0189 | hypothetical protein | **-2.228312** |
| A1S_3046 | oligopeptidase A | **-2.226816** |
| A1S_2092 | aminopeptidase N | **-2.222411** |
| A1S_0204 | class II aldolase/adducin domain-containing protein | **-2.216437** |
| A1S_0959 | signal peptide | **-2.185125** |
| A1S_0200 | inorganic pyrophosphatase | **-2.184551** |
| A1S_1670 | secretion protein HlyD | **-2.172889** |
| A1S_2099 | hypothetical protein | **-2.171868** |
| A1S_0184 | hypothetical protein | **-2.153121** |
| A1S_2334 | S-adenosyl-L-homocysteine hydrolase | **-2.145865** |
| A1S_0426 | hypothetical protein | **-2.138868** |
| A1S_0196 | like acyl-CoA synthetase | **-2.130694** |
| A1S_2764 | tRNA-Arg | **-2.128246** |
| A1S_1341 | enoyl-CoA hydratase/carnithine racemase | **-2.127496** |
| A1S_2103 | transport protein | **-2.119765** |
| A1S_0651 | TraB protein | **-2.106580** |
| A1S_2749 | hypothetical protein | **-2.104598** |
| A1S_1122 | short-chain dehydrogenase | **-2.091194** |
| A1S_2623 | hypothetical protein | **-2.079956** |
| A1S_2888 | hypothetical protein | **-2.075166** |
| A1S_1120 | lipase/esterase | **-2.070525** |
| A1S_0177 | cysteine synthase A/O-acetylserine sulfhydrolase A subunit PLP-dependent enzyme | **-2.067469** |
| A1S_1314 | ankyrin repeat-containing protein | **-2.065234** |
| A1S_0070 | D-lactate dehydrogenase | **-2.051314** |
| A1S_0977 | arylsulfatase | **-2.049950** |
| A1S_3159 | lipase chaperone | **-2.049089** |
| A1S_0953 | hypothetical protein | **-2.046882** |
| A1S_1365 | phosphonoacetaldehyde hydrolase | **-2.039098** |
| A1S_2122 | transcriptional regulator | **-2.038113** |
| A1S_1133 | flavin-binding monooxygenase | **-2.033929** |
| A1S_1787 | iron transport protein | **-2.027750** |
| A1S_0172 | hypothetical protein | **-2.020550** |
| A1S_0029 | ABC-type nitrate/sulfonate/bicarbonate transport systems | **-2.013934** |
| A1S_1423 | malonate decarboxylase subunit delta | **-2.009808** |
| A1S_0149 | membrane-bound ATP synthase F0 sector, subunit a | **-2.000177** |

**Table S2. Effect of LeuO deletion on antibiotic susceptibility**

| **Antimicrobial agents** | **MIC (µg/mL)** | | |  |
| --- | --- | --- | --- | --- |
|  | **Wild-Type** | **∆LeuO** | **CP** | **Fold change** |
| Ceftazidime | 8 | 8 | 8 | 1 |
| Meropenem | 4 | 2 | 2 | 2 |
| Imipenem | 0.5 | 0.25 | 0.5 | 2 |
| Aztreonam | 32 | 32 | 32 | 1 |
| Amikacin | 4 | 2 | 1 | 2 |
| Gentamicin | 2 | 1 | 2 | 2 |
| Tobramycin | 2 | 0.5 | 0.5 | 4 |
| Tetracycline | 2 | 2 | 2 | 1 |
| Ciprofloxacin | 0.25 | 0.25 | 0.25 | 1 |
| Levofloxacin | 0.5 | 0.25 | 0.5 | 2 |

| **Table S3. Bacterial strains and plasmids used in this study.** | | |
| --- | --- | --- |
| **Bacteria or plasmids** | **Relevant characteristics** | **Reference or source** |
| **Bacterial strains** |  |  |
| ***A. baumannii*** |  |  |
| ATCC 17978 | Wild-type strain | Lab stock |
| ΔA1S_1874 | ATCC 17978 with ΔA1S_1874 | This study |
| Complementary strain | A1S_1874 rescue in ΔA1S_1874 | This study |
| 1656-2 | Wild-type strain | Laboratory collection |
| ST-208(20132411)  (011) | Clinical strain | Laboratory collection |
| ST-229(20145719)  (079) | Clinical strain | Laboratory collection |
| ST-357(20130721) ((004) | Clinical strain | Laboratory collection |
| ST-451(KBN10P04625) (001) | Clinical strain | Laboratory collection |
| ST-552(20133395)  (015) | Clinical strain | Laboratory collection |
| ST-784(KBN10P04703) (001) | Clinical strain | Laboratory collection |
| ***E. coli*** |  |  |
| SY327 λ *pir* | *supE44* Δ*lacU169* (ф80 *lacZ*ΔM15*) hsdR17*  *recA1 endA1 gyrA96 thi-1 relA1 λpir* (phage  lysogen); plasmid replication | Laboratory collection |
| SM10 λ *pir* | *thi thr leu tonA lacY supE recA::RP4-2-*  *Tc::Mu Km λpir* π-requiring plasmids; conjugal donor | Laboratory collection |
| DH5α *λ pir* | *supE44 DlacU*169 *(f80 lacZ DM15) hsdR17 recA1 endA1 gyrA 96 thi-1 relA1 λ* pir phage lysogen; plasmid replication | Laboratory collection |
| *E. coli* ATCC 25922 | Quality control strain for MIC | Laboratory collection |
| *P. aeruginosa* ATCC 27853 | Quality control strain for MIC | Laboratory collection |
| **Plasmids** |  |  |
| pOH4 | pHKD01 with *ompA* coding region with *nptI*; Km^r^ | Laboratory collection |
| pDM4 | Suicide vector; *ori*R6K, *sacB*, and Cm^r^ | Laboratory collection |
| pDM4::ΔA1S_1874 | pDM4 with ΔA1S_1874::*nptI*; , Cm^r^, Km^r^ | This study |
| pDM4::A1S_1874(T1) | pDM4 with A1S_1874 (T1 terminator)::*nptI*; , Cm^r^, Km^r^ | This study |

Tri^R^, trimethoprim resistant; Cm^R^, chloramphenicol resistant; Km^R^, kanamycin resistant

**Table S4. Oligonucleotides used in this study.**

| **Primers** | **Sequence (5’ to 3’)** |
| --- | --- |
| Construct for A1S_1874 knockout strain | |
| A1S_1874 up F  (Gibson assembly) | CGGATAACAATTTGTGGAATCCC CCGCTCCACCAATAAAATTTTCAAG |
| A1S_1874 up R | CTTAATAGATCAATTCGCTTTACATGAGGTGTTATTTAATAAAATCATTTCATTC |
| A1S_1874 down F | CACCTCATGTAAAGCGAATTGATCTATTAAG |
| A1S_1874 down R | CACCTTCTTCACGAGGCAGACGCAACCACTTTAAAGTCTTGTCC |
| NptⅠ F | GTCTGCCTCGTGAAGAAGGTG |
| NptⅠ R  (Gibson assembly) | GGTAACCTGAGCTCTCCCGATCCGTCGACCTGCAGG |
| Construct for A1S_1874 complementary strain | |
| glms up F SpeⅠ | GGA CTA GTT GGT TTG AGC AAT TGA CTT GG |
| glms up R | GTTTTTACCCGAGCCTTAATAATGATCTTTTTTGAATTACTCTACAGT |
| A1S_1874 F | AAAGATCATTATTAAGGCTCGGGTAAAAACATATTCCAAAGACC |
| A1S_1874 R | TTATTTGATGCCTTACTGAAAAAGTTTAATAATTTCATGTCGCAAAAA |
| T1 terminator F | ACTTTTTCAGTAAGGCATCAAATAAAACGAAAGGCTCA |
| T1 terminator R | CAAATCCGCCGCCCTAGAATCACCTGCTTTA |
| glms down F | CGCCCTAGAATCACCTGCTTTAATAATTGATTGATTAAGCC |
| NptⅠ R ApaⅠ | GGGCCCGATCCGTCGACCTGCAGG |
| glms up seq F | TGG CGA AGT CAG TAA CTG TAG A |
| glms down seq R | GGA AAG CTT CTG TGT AGC GAT ATG |
| Real time PCR primers | |
| 16S rRNA-sense | ACT CCT ACG GGA GGC AGC AGT |
| 16S rRNA-antisense | TAT TAC CGC GGC TGC TGG C |
| CsuC-sense | AAAGCAGGCGAGAAGCATATG |
| CsuC-antisense | GGATCGGCAACTCATCTACAATC |
| CsuD-sense | ACCCTATCAAGGCGGTTCAAC |
| CsuD-antisense | CGACGATAGCCGTCATTATCTACA |
| CsuE-sense | TCAGACCGGAGAAAAACTTAACG |
| CsuE-antisense | GCCGGAAGCCGTATGTAGAA |
| A1S_0112-sense | CGAATGCTCTGTTGAAATGG |
| A1S_0112-antisense | ACCCCTTCAGAATCAGCAAA |
| A1S_0117-sense | TCCTGAATCTGGAACCAATAAATG |
| A1S_0117-antisense | ACGCAATGGAATCACATCTGAA |
| A1S_0118-sense | AGGTTTTATTGGCAGCAGATTGA |
| A1S_0118-antisense | TCTTGGTTGCGGCATTACG |
| A1S_1772-sense | AGGCTTAGGCTGGGTAAAACTTC |
| A1S_1772-antisense | GGATTGCTTTTATGGTCTGTGTTG |
| A1S_1773-sense | ACGACCGTGCAGACTTTTCAT |
| A1S_1773-antisense | CCGTTCTGTGGTCAGCCTAAA |
| A1S_2304-sense | TGGGACTTGCCAATGAAACAG |
| A1S_2304-antisense | GTTGTATTCAGGTTGTTATCGATTGAG |
| A1S_2305-sense | GGAATCTTAGCGGCTCTGACA |
| A1S_2305-antisense | CCGATTTGGGTAAAGATGTTGTTA |
| A1S_1063-sense | CACGCCACCAGCTAAATCAAG |
| A1S_1063-antisense | TGAGCGTGGTAACCCAAAAAG |
| A1S_0242-sense | GGAAGTGATTACCAAGGGTGTTTT |
| A1S_0242-antisense | AAGCGAAACGAGTAAAGCCAAT |
| A1S_0628-sense | GCACTCCTGAGTCGTGGAAATAT |
| A1S_0628-antisense | TTGATTTCGCCCTTGTTTGC |
| A1S_1256-sense | TTCGTACCGCTATTCGTTTGTTTA |
| A1S_1256-antisense | TTCGGTATTTCTGATTCTTTGACAAT |
| A1S_1292-sense | AAACGAGTAGTAAGTCCAGCAAAGG |
| A1S_1292-antisense | AGGCGAACTTTTTGATTTATTGACA |
| A1S_1295-sense | GTTGAAGGTGGTGGTTTAGTTGAA |
| A1S_1295-antisense | AATACCACTTCGCCATCTTGTGT |
| A1S_1385-sense | GGCGCTTGTCGGTCAGTTAA |
| A1S_1385-antisense | TCGGGATTTGATAACCATTGATT |
| A1S_2347-sense | TTCTTTGCCCGTCCTGTTATTAC |
| A1S_2347-antisense | GCTAGAACATGATAACTTGGGAAACTC |
| A1S_0644-sense | TAAGGCGTCCACACCGATTT |
| A1S_0644-antisense | TCTCACATACCCCACCAAACTG |
| A1S_0645-sense | AACAAGGGCAGGGTCAAGGT |
| A1S_0645-antisense | CGGTAGCGGTAAGAGGTTCAA |
| A1S_0800-sense | ATGCGTGGCAATCCAGAAGT |
| A1S_0800-antisense | ATCACGGGCAGCCAACTCT |
| A1S_1288-sense | AGTGGTGGGTTTAGAGGGTGAA |
| A1S_1288-antisense | CGGGTAAAAGGAAACGGACTT |
| A1S_3364-sense | GTCCCACGACTTTCGCTCTTT |
| A1S_3364-antisense | ACGGCACCTGAATATAATCCAGAA |

Bhargava, N., Singh, S.P., Sharma, A., Sharma, P., and Capalash, N. (2015). Attenuation of quorum sensing-mediated virulence of Acinetobacter baumannii by Glycyrrhiza glabra flavonoids. Future Microbiol 10, 1953-1968.

Jung, H.W., Kim, K., Islam, M.M., Lee, J.C., and Shin, M. (2020). Role of ppGpp-regulated efflux genes in Acinetobacter baumannii. J Antimicrob Chemother 75, 1130-1134.

Park, J.Y., Kim, S., Kim, S.M., Cha, S.H., Lim, S.K., and Kim, J. (2011). Complete genome sequence of multidrug-resistant Acinetobacter baumannii strain 1656-2, which forms sturdy biofilm. J Bacteriol 193, 6393-6394.
